# Quantitative Evaluation of Thermomechanical Fatigue Damage in Composite Materials Using X-Ray Computed Tomography and Machine Learning

**DOI:** 10.3390/ma19173752

**Published:** 2026-09-03

**Authors:** Piotr Przystałka, Paweł Chrzanowski, Tomasz Rogala, Andrzej Katunin

**Affiliations:** Department of Fundamentals of Machinery Design, Faculty of Mechanical Engineering, Silesian University of Technology, Konarskiego 18A, 44-100 Gliwice, Poland; piotr.przystalka@polsl.pl (P.P.); pawel.chrzanowski@polsl.pl (P.C.); tomasz.rogala@polsl.pl (T.R.)

**Keywords:** damage quantification, thermomechanical fatigue, self-heating effect, polymer-matrix composites, X-ray computed tomography, non-destructive testing, machine learning, classification, segmentation

## Abstract

The fatigue degradation of polymer-matrix composite materials, especially in the case of thermomechanical fatigue, when the self-heating effect dominates the process of the cyclically bent structure in fully reversed mode, results in complex damage patterns with a heterogeneous nature. It is therefore essential to evaluate the structural condition of tested objects in a non-destructive way to predict their fatigue lifetime. One of the most accurate non-destructive testing techniques used for such examinations is X-ray computed tomography, which enables the evaluation of the resulting fatigue damage in a three-dimensional space. However, the main challenge in the evaluation of such results is the attribution of the observed damage to specific types, the assessment of its severity, and its quantitative analysis. In this study, we have proposed a novel framework based on intensity and morphological features for the classification of damage types and a 3D U-Net-based approach for segmentation. The acquired results demonstrated the high accuracy of the performance evaluation indices, which exceeded 99% for the proposed framework, allowing effective evaluation of the resulting damage and being potentially helpful in the assessment of structural performance and residual fatigue life. Such analysis contributes to the trends of NDE 4.0 and adaptive mechanics, where composite structures are tailored to specific operational conditions to reach the target lifecycle.

## 1. Introduction

In light of recently implemented safety requirements for composite structures, primarily in the aviation and automotive industries, the periodic non-destructive evaluation (NDE) of such structures is an inevitable element of their maintenance. This is confirmed by appropriate regulations defined by the damage tolerance and condition-based maintenance design and operation philosophies being used nowadays for aircraft structures [1,2,3,4,5]. The application of NDE implies a possibility of tracking changes in structures in operation and, based on them, predicting and prognosing their structural residual life. A great variety of non-destructive testing (NDT) techniques opens a wide range of possibilities for the NDE of structures with various sensitivities to particular types of defects and damage, availability for testing, materials and geometric properties of tested structures, and many other factors. An overview of the performance of various NDT techniques for composite structures can be found, e.g., in [6].

Due to the mentioned sensitivity factors that determine the selection of the appropriate NDT technique, in numerous cases, the only possible solution that provides satisfactory accuracy of the results is X-ray computed tomography (XCT). This technique has numerous limitations, including the dimensions of a tested object or structure, desired material properties, duration of testing, high inspection costs, etc.; however, it provides NDT results in the form of a 3D array with superior resolution, and, therefore, it is often selected also for the inspection of composite structures [6,7,8,9]. Besides the numerous advantages mentioned in the above-cited studies, the problem of the proper interpretation of scanning results still exists. This is mostly due to the high complexity of the internal architecture of polymer-matrix composite (PMC) materials and, therefore, the high complexity of resulting XCT scans, but also to the presence of scanning artifacts, which additionally complexify the proper recognition of damaged areas [10]. This deficiency makes it difficult to automatize inspection. However, numerous attempts to overcome this deficiency were undertaken in various applications [11]. From the point of view of the improvement and automation of XCT data processing, this implies the necessity of developing two classes of tools: post-processing algorithms, which distinguish damaged and healthy areas in tested structures; and recognition algorithms, which perform classification of types of detected damage.

Several attempts have been made in the development of both types of such algorithms. The post-processing of XCT data is usually performed using image segmentation techniques, which reveal good results in cases when the damage is remarkably distinguishable with respect to healthy material [12,13,14,15,16,17,18,19]. In a number of cases, the tomograph-dedicated software is enough to properly segment XCT data for more complex structures [20,21]. However, in the case of PMCs, basic segmentation methods are ineffective. This is due to the presence of textures (e.g., in the form of reinforcement), insignificant differences between the damaged and undamaged areas of a structure, the presence of artifacts, etc. The image segmentation of XCT results using the seed-growth approach was proposed by Bull et al. [7]. An advanced segmentation approach for XCT scans was proposed by Jørgensen et al. [22], where they used level set methods for the iterative segmentation of defects. Another segmentation procedure to isolate voids from 2D slices of XCT scans of PMCs was presented by Scott et al. [23]. Recently, the segmentation approach based on the statistical distributions and probability analysis of identified colors in XCT scans was proposed in [24]. In particular, the authors of [25] used XCT for surface extraction from multi-material components using image processing and fusion techniques, and the authors of [7] used 3D segmentation techniques for the extraction and classification of impact damage, while the authors of previous studies were focused on distinguishing damage types using 3D wavelet transform [26], detecting the directionality of delaminations in PMCs using a combination of Hough and wavelet transforms [27], and distinguishing fatigue damage types using advanced image-processing algorithms [28]. Overviews of approaches applied to image segmentation from XCT scans that discuss numerous factors influencing segmentation accuracy can be found in [29,30].

The second class of tools used for supporting the NDE of PMCs is oriented to the classification of different types of damage. Numerous approaches developed to date are based on the evaluation of the morphology of structural damage to classify types of damage (various types of cracks, delamination, voids, foreign object inclusions, etc.; see [31] for a description of damage appearing in PMCs). This approach was used by Jac Fredo et al. [32], where the authors considered geometric features and Zernike moments to further classify damage in PMCs using the support vector machine algorithm. The algorithms based on morphological features (dimensions, dimension ratios, orientation) were previously used to classify damage in PMCs by the authors’ team [24,26,28]. In numerous cases, the classification step is connected with pre-processing procedures, including segmentation, as was demonstrated in previous studies. Several examples of such a connection can be found in the literature; see, e.g., [33,34,35].

However, the use of XCT for quantitative damage evaluation is strongly dependent on the quality of the image segmentation. Segmentation converts grayscale tomographic data into discrete material or damage classes and is therefore a critical step before calculating damage volume, crack density, connectivity, spatial distribution, or damage evolution indicators. In PMCs, this task is particularly challenging because fibers, polymer matrix, and damage regions often exhibit low gray-level contrast, while XCT volumes may contain noise, ring artifacts, beam-hardening effects, partial-volume effects, and local intensity inhomogeneities [10,36]. Conventional segmentation methods based on global or local thresholding, morphological operations, or handcrafted filters can be effective in relatively simple cases, but their performance decreases when the target defects are small, discontinuous, poorly contrasted, or morphologically similar to image artifacts [36,37]. Moreover, such methods usually require expert-dependent parameter tuning, which limits repeatability and scalability when large three-dimensional datasets are analyzed.

In the problem of structural damage classification, recent attempts have been made using machine learning (ML) techniques. One of the most popular approaches is based on the classification of damage using artificial neural networks (ANNs). Such an approach was implemented by Stamopoulos et al., who used ANNs for the classification of pores in carbon fiber-reinforced polymer composites based on collected XCT scans [38]. Recent developments in ML bring new comprehensive tools, in particular deep learning techniques, which define a new level of classification performance in numerous applications.

The concept of convolutional neural networks (CNNs) has also found numerous applications in problems related to damage detection, identification, and classification, in particular, based on XCT scans. Based on such an approach, Sammons et al. [33] performed automatic detection of delamination in PMCs from XCT scans. Hu et al. [39] applied CNNs for automatic detection of defects in the aluminum conductor composite core used in electric power transmission problems. Recent studies by Gong et al. [40] demonstrated an application of CNNs to the detection of inclusions and voids in spacecraft composite structures. The mentioned applications reveal very high performance in the identification and classification of structural damage, which confirms the purposefulness of using CNNs in the investigated problem. Sinchuk et al. compared variational and deep learning-based approaches for very-low-contrast micro computed tomography (µCT) images of carbon/epoxy woven composites and showed that U-Net-based segmentation can be a useful alternative when the contrast between fiber and polymer is insufficient for robust conventional segmentation [36]. Bertoldo et al. proposed a modular U-Net framework for automated segmentation of XCT images in composite materials and demonstrated that U-Net-based models can support automated processing of complex three-dimensional tomography datasets with a limited number of annotated slices [41]. Badran et al. emphasized that deep learning segmentation of XCT images of fiber-reinforced PMCs requires careful validation against reference data, as visual inspection alone is insufficient to quantify detection performance or measurement accuracy [42]. These studies indicate that CNNs can reduce the dependence on manual interpretation and support the quantitative analysis of XCT datasets.

Recent studies have also begun to apply deep learning directly to damage-related segmentation tasks. Helwing et al. used a CNN-based approach for the analysis and segmentation of fatigue damage mechanisms in X-ray CT data of fiber-reinforced polymers, allowing a quantitative mechanism-differentiated assessment of matrix cracks, interfiber failure, and delamination [43]. Guo et al. combined deep-learning-based image enhancement with U-Net-type segmentation to improve fiber segmentation in time-resolved tomography of fiber-reinforced PMCs, showing that the quality of the tomographic data and the segmentation model should be considered jointly [44]. Petkov et al. used an Attention U-Net architecture for semantic segmentation of progressive transverse micro-cracking in PMCs and showed that attention mechanisms may improve segmentation of selected crack classes in challenging tomographic data [45]. Wang et al. integrated in situ XCT, U-Net-based semantic segmentation, and digital volume correlation to characterize damage initiation and propagation in woven CFRPs [46]. These works confirm that deep learning is becoming an important tool for extracting quantitative information from XCT datasets of PMCs.

From a methodological perspective, the XCT-based approaches reviewed above can be broadly divided into three groups. The first group comprises object-level workflows in which damage regions are extracted and subsequently characterized or classified using handcrafted morphological, geometrical, or intensity-based descriptors [19,32,47]. Their main advantage is the interpretability of the resulting component-level description, whereas their principal limitation is the dependence of the final classification on the quality of the preceding segmentation and component-extraction stage. The second group comprises deep learning-based semantic segmentation methods, including U-Net-based approaches [36,41,43,45], which provide spatially resolved damage maps and exploit image context directly, but require reliable reference annotations and generally provide less direct object-level interpretability. A third group combines deep-learning segmentation with subsequent quantitative or mechanics-oriented analysis, for example, by coupling semantic segmentation with digital volume correlation [46]. Such approaches extend segmentation beyond damage localization, but do not provide the same type of interpretable component-level damage classification considered in feature-based workflows. Thus, within the literature reviewed above, object-level damage classification and voxel-wise deep learning segmentation are predominantly investigated as separate analytical routes or combined with different downstream analyzes, rather than evaluated as complementary descriptions of the same fatigue-damage problem within a common XCT dataset.

An additional limitation of the existing literature is that object-level classification and deep-learning segmentation are generally reported for different datasets, material systems, damage mechanisms, or XCT acquisition conditions. Consequently, their relative advantages cannot be assessed directly without the confounding influence of differences in the underlying experimental data. Therefore, a controlled evaluation using a common source dataset is particularly valuable when the objective is to determine how component-level classification and voxel-wise segmentation complement each other in quantitative fatigue-damage assessment. To the best of the authors’ knowledge, the complementary application of interpretable object-level feature-based classification and volumetric deep learning segmentation to the quantitative assessment of thermomechanical fatigue damage has not previously been systematically investigated within a common experimental XCT dataset. This is especially relevant for fatigue-induced damage, where the target regions are sparse, small, and strongly imbalanced with respect to the background material. Moreover, in many cases the final interpretation of inspection results should be performed by a human, i.e., an inspector, which makes this process extremely demanding. In numerous industries, like aircraft and nuclear, the inspection process cannot be fully automated due to the great responsibility for the safety and integrity of the structures or objects being evaluated. However, due to the above-mentioned uncertainties and taking into account uncertainties generated by an inspector during the interpretation of NDE results (e.g., possible overlooking of important information), there is a need to support NDT inspectors with developed tools, which will allow us to extract relevant NDT data and be helpful in the decisive process. Supporting the decisive process has numerous economic benefits. Firstly, due to the large amount of inspection data, the automation of this process (or at least its main part) can significantly shorten the evaluation duration, allowing for the shortening of very costly man-hours of high-level NDT inspectors, as well as reducing the duration of the maintenance process. Secondly, decision-supporting systems can be implemented in such a way that low-level NDT inspectors perform inspections, while the high-level NDT inspector collects NDT data remotely and automatically pre-processes it, which falls into the currently developed strategies within the fourth industrial revolution [48], including the combination of NDT with the Internet of Things [49].

Therefore, the present study addresses a gap in the quantitative identification and classification of fatigue-induced damage in PMCs from XCT data. Unlike approaches that focus primarily on the overall fatigue response or on the detection of a single damage type, the present work considers the simultaneous identification of multiple damage classes characterized by small dimensions, sparse occurrence, irregular morphologies, and heterogeneous spatial distributions within the composite volume. These material-specific characteristics pose a particular challenge to reliable damage characterization in volumetric XCT data. To address this gap, the present study develops two complementary analytical routes using the same underlying collection of XCT cases. At the object level, the extracted damage components are classified using machine learning models based on a fused representation of three-dimensional morphological and XCT intensity descriptors. At the voxel level, a 3D U-Net performs multiclass volumetric segmentation to determine the spatial distribution and extent of delamination- and crack-like damage in full XCT volumes. Importantly, these two routes do not constitute a serial classification–segmentation cascade. Instead, they provide parallel and complementary descriptions of the same damage problem and are evaluated using a common experimental XCT source dataset and the same independent case-level holdout subset. This design enables the capabilities and limitations of interpretable component-level classification and spatially explicit volumetric segmentation to be assessed under consistent material, thermomechanical degradation, XCT acquisition, and holdout conditions. Therefore, the methodological contribution of the present study lies not only in combining morphological and intensity information within the FUSED feature representation and in applying a dedicated 3D U-Net segmentation workflow, but also in evaluating both analytical levels within one consistent XCT dataset. This provides complementary information on damage type, morphology, spatial distribution, and extent while avoiding differences between datasets as a confounding factor when interpreting the two analytical routes. For the segmentation route, the proposed approach additionally exploits three-dimensional spatial context, class-aware sampling of volumetric patches, domain-specific augmentation, and a foreground-oriented loss formulation to address the sparse and strongly imbalanced nature of fatigue-induced damage.

## 2. Theoretical Background on Classification and Segmentation

### 2.1. Feature-Based Classification of XCT Damage Components

Feature-based classification constitutes an object-level approach for the quantitative analysis of damage identified in volumetric XCT data. This approach operates on three-dimensional components representing isolated damage regions. Each detected component is treated as an independent observation and classified into one of the predefined damage categories. This formulation provides a physically interpretable representation of fatigue damage and allows quantitative comparison of different machine learning algorithms operating on tabular feature data [19,32,38].

Let

(1) $$C_{i}=\{v_{1},v_{2},\ldots ,v_{n_{i}}\},$$

denote the i-th connected component composed of $n_{i}$ voxels. Each component is represented by a numerical feature vector

(2) $$x_{i}=[f_{1}\left(C_{i}\right),f_{2}\left(C_{i}\right),\;\ldots ,\;f_{p}\left(C_{i}\right){]}^{T},$$

where $f_{j}(C_{i})$ denotes the j-th feature extracted from the component and p is the total number of features. For each component, the corresponding class label was assigned as:

(3) $$y_{i}\;\in \;\{D1,\;D2\},$$

where D1 corresponds to delamination, and D2 corresponds to crack damage. The classification problem can, therefore, be formulated as a mapping

(4) $$f\;:R^{p}\to \{D1,\;D2\},$$

which assigns a damage class to the feature vector describing an individual component.

#### 2.1.1. Preprocessing, Features’ Representation and Selection

The discriminatory capability of the classifier depends directly on the adopted feature representation. In the proposed framework, each component is characterized by complementary XCT intensity-based features and three-dimensional morphological features. XCT intensity-based features quantify the distribution of grayscale intensities within the damage region and its local neighborhood in the XCT volume, whereas morphological features characterize the size, compactness, spatial extent, and shape of the extracted component. The combined representation enables discrimination between defects exhibiting similar grayscale-intensity characteristics but substantially different three-dimensional morphology.

The mean grayscale intensity of a component is defined as

(5) $$\mu_{i}=\frac{1}{n_{i}}\sum_{v\in C_{i}}I(v),$$

where I(v) denotes the grayscale intensity assigned to voxel v. The corresponding standard deviation is calculated as

(6) $$\sigma_{i}=\sqrt{\left(\left(\frac{1}{n_{i}-1}\right)\sum_{v\in C_{i}}{\left(I\left(v\right)-\mu_{i}\right)}^{2}\right)},\;$$

which quantifies local intensity variability within the component. To evaluate the visibility of a damage component with respect to its surrounding material in the XCT data, the local contrast is defined as

(7) $${LC}_{i}=\mu_{i}-\mu_{i}^{B},$$

where $\mu_{i}^{B}$ denotes the mean grayscale intensity of the local background. In this study, the local background means the non-component neighborhood surrounding a given component. The local contrast-to-noise ratio is then expressed as

(8) $${CNR}_{i}=\frac{\left|{LC}_{i}\right|}{\sqrt{\left[\left(\sigma_{i}^{2}+{\left(\sigma_{i}^{B}\right)}^{2}\right)/2\right]}}\;,$$

where $\sigma_{i}^{B}$ denotes the standard deviation of grayscale intensities calculated for the local background. This feature combines contrast information with local intensity variability and, therefore, provides a normalized measure of component visibility in XCT data.

The morphological feature group includes component volume, surface area, three principal-axis lengths, three-dimensional solidity, circumferential diameter, inscribed-sphere diameter, and sphericity-related features, namely WadellSphericity, TickellSphericity, RilleySphericity, CoxBudhuSphericity, and TrueSphericity. These features characterize the size, spatial extent, compactness, local thickness, and shape regularity of individual components without imposing a single specific definition of sphericity. Such a formulation is suitable for fatigue damage in polymer-matrix composites, because delamination and crack components may exhibit partially similar grayscale-intensity characteristics while differing considerably in their three-dimensional morphology.

The resulting feature vectors constitute the input to supervised machine learning classifiers operating in multidimensional feature space. Due to the high number of the considered features, feature selection is performed using the minimum Redundancy Maximum Relevance (mRMR) criterion, which seeks features exhibiting high relevance to the target classes while minimizing redundancy among the selected variables [50].

#### 2.1.2. Component-Level Classification Metrics

The performance of feature-based classifiers is evaluated using metrics derived from the binary confusion matrix. In the considered problem, class D2, which corresponds to crack damage, is treated as the positive class, whereas class D1, which corresponds to delamination, is treated as the negative class. Therefore, TP denotes the number of correctly classified crack components, FP denotes delamination components incorrectly classified as cracks, FN denotes crack components incorrectly classified as delamination, and TN denotes correctly classified delamination components.

The overall classification accuracy is defined as

(9) $$Accuracy=\frac{TP+TN}{TP+TN+FP+FN}.$$

For the minority crack class D2, precision and recall are calculated as

(10) $$Precision_{D2}=\frac{TP}{TP+FP}\;,$$

and

(11) $${Recall}_{D2}=\frac{TP}{TP+FN}\;,$$

respectively. $Precision_{D2}$ describes the reliability of crack predictions, whereas $Recall_{D2}$ describes the sensitivity of the classifier to actual crack components. The harmonic mean of these two quantities is expressed by the F1-score:

(12) $$F1_{D2}=\frac{2\cdot Precision_{D2}\cdot Recall_{D2}\;}{\left(Precision_{D2}+Recall_{D2}\right)}.$$

In addition to threshold-dependent metrics, ranking-based measures are also considered. The ROC-AUC represents the area under the receiver operating characteristic curve, which relates the true-positive rate to the false-positive rate over different classification thresholds. The PR-AUC represents the area under the precision–recall curve and is particularly informative for the minority crack class under class-imbalanced conditions. The combined use of Accuracy, Precision for D2, Recall for D2, F1-score for D2, ROC-AUC and PR-AUC provides a complete assessment of classifier performance presented for classification in this study. It should be emphasized that these metrics quantify the predictive performance of the component-level classifiers, not the physical evolution of thermomechanical fatigue degradation. They assess how effectively the classifiers distinguish between the D1 and D2 components in the XCT-derived dataset. They do not describe degradation kinetics, fatigue-life evolution, damage-growth rate, or changes in residual mechanical properties. Quantifying such material-level relationships would require a separate analysis linking XCT-derived damage measures with loading history, self-heating temperature evolution, and mechanical performance indicators.

### 2.2. Volumetric Semantic Segmentation for XCT Damage Analysis

In addition to the classification task discussed in the preceding subsection, this study also considers semantic segmentation of fatigue-induced damage in three-dimensional XCT volumes, which provides a spatially explicit formulation of the damage evaluation problem. While classification assigns a label to a sample, slice, or region, segmentation assigns a damage class to each voxel of the reconstructed volume. This distinction is essential for the present study, because the objective is not limited to detecting whether damage is present, but also includes determining its spatial distribution, morphology, and extent. Therefore, semantic segmentation provides the basis for a quantitative evaluation of internal fatigue damage in composite materials.

The segmentation task considered in this work is formulated as a multiclass volumetric prediction problem. Let

(13) $$X\in R^{H\times W\times D\times 1}$$

denote a grayscale XCT volume or a three-dimensional grayscale patch extracted from it, where *H*, *W*, and *D* are the spatial dimensions, and the final singleton dimension represents the single intensity channel. The aim is to estimate a dense label map

(14) $$\hat{Y}\in C^{H\times W\times D},$$

where C is the set of segmentation classes. In the proposed workflow, the multiclass representation is defined as C={D0,D1,D2}, where class D0 denotes background or non-damaged regions, class D1 denotes delamination-like damage, and class D2 denotes crack-like damage. This formulation was selected because fatigue degradation in composites is not a single homogeneous phenomenon. Separating delamination-like and crack-like regions allows the segmentation results to be interpreted with respect to different damage morphologies and allows for class-specific quantitative evaluation.

#### 2.2.1. Semantic Segmentation of Three-Dimensional XCT Volumes

In the present work, semantic segmentation is treated as a dense voxel-wise prediction in the full three-dimensional XCT domain. This is important because fatigue damage may be weakly visible or ambiguous in individual two-dimensional slices, whereas its continuity, elongation, and spatial relation to neighboring structures are better represented in the volumetric domain. Slice-wise processing may therefore lose relevant information about the three-dimensional geometry of cracks and delamination-like regions. For each voxel i, the segmentation network estimates a vector of class scores

(15) $${\hat{p}}_{i}=\left[{\hat{p}}_{i,0},{\hat{p}}_{i,1},{\hat{p}}_{i,2}\right],$$

where

(16) $$\sum_{c\in C}{\hat{p}}_{i,c}=1.$$

The raw voxel label is obtained from the maximum class score:

(17) $${\hat{y}}_{i}^{raw}=\arg\underset{c\in C}{max}\;{\hat{p}}_{i,c}.$$

However, in the XCT damage segmentation problem considered, the foreground classes represent small and sparse regions. For this reason, class-dependent acceptance thresholds can be applied to reduce isolated false positive responses:

(18) $${\hat{y}}_{i}=\left\{\begin{array}{ll} {\hat{y}}_{i}^{raw}, & {\hat{p}}_{i,{\hat{y}}_{i}^{raw}}\geq \tau_{{\hat{y}}_{i}^{raw}},\\ 0, & {\hat{p}}_{i,{\hat{y}}_{i}^{raw}}<\tau_{{\hat{y}}_{i}^{raw}}, \end{array}\right.$$

where $\tau_{c}$ is the minimum acceptance score for class c. In this article, this mechanism is used as part of the final voxel-label assignment for damage classes, while the detailed parameter values are reported in Section 3.

#### 2.2.2. 3D U-Net for Dense Volumetric Prediction

The dense segmentation model used in this work is based on the 3D U-Net architecture, which extends the encoder–decoder U-Net concept to volumetric data [51,52]. This architecture is suitable for XCT damage analysis because it combines hierarchical feature extraction with dense spatial reconstruction. The encoder captures increasingly abstract volumetric features, whereas the decoder restores the original spatial resolution required for voxel-wise prediction. Skip connections transfer high-resolution information from the encoder to the decoder, which is important for preserving small cracks and thin delamination-like regions.

For an input tensor A at level l, a 3D convolutional block can be written as

(19) $$F_{l}\left(A\right)=\phi \left(K_{l,2}*\phi \left(K_{l,1}*A+b_{l,1}\right)+b_{l,2}\right),$$

where ∗ denotes 3D convolution, $K_{l,1}$ and $K_{l,2}$ are convolution kernels, $b_{l,1}$ and $b_{l,2}$ are bias terms, and $\phi \left(\cdot \right)$ is a nonlinear activation function. In the encoder path, the feature maps are computed as

(20) $$E_{l}=F_{l}^{enc}\left(X_{l}\right),$$

followed by spatial downsampling:

(21) $$X_{l+1}=P\left(E_{l}\right),$$

where $P\left(\cdot \right)$ denotes a 3D pooling operation. In the decoder path, the upsampled representation is concatenated with the corresponding encoder features:

(22) $$S_{l}=concat\left(U\left(D_{l+1}\right),E_{l}\right),$$

where $U\left(\cdot \right)$ denotes upsampling or transposed convolution and $D_{l+1}$ is the decoder representation from the lower-resolution level. The output layer maps the decoder features to class logits:

(23) $$z_{i,c}={\left(K_{c}^{out}*D_{0}\right)}_{i}+b_{c}^{out},$$

where $z_{i,c}$ is the logit for voxel i and class c. The normalized class score is obtained using the softmax function:

(24) $${\hat{p}}_{i,c}=\frac{exp\left(z_{i,c}\right)}{\sum_{k\in C}exp\left(z_{i,k}\right)}.$$

In the context of this article, the use of 3D convolutions is not merely a technical extension of a two-dimensional model. It is required to exploit the volumetric continuity of damage and distinguish spatially coherent defects from local artifacts or noise.

#### 2.2.3. Class Imbalance and Foreground-Oriented Loss Functions

A characteristic feature of XCT fatigue-damage segmentation is the strong class imbalance between the background and damage classes. Most voxels belong to non-damaged material, while crack- and delamination-like regions occupy only a small fraction of the reconstructed volume. If this imbalance is not considered, the model may obtain a low global error by favoring the dominant background class while still missing small but physically important damage regions.

To address this issue, the proposed training objective combines weighted cross-entropy with a foreground-oriented Tversky loss. Let $n_{c}$ denote the number of voxels of class c in the training set and

(25) $$f_{c}=\frac{n_{c}}{\sum_{k\in C}n_{k}},$$

be the empirical class frequency. In the implementation, classes with zero voxel count are first assigned a count of one to avoid undefined frequencies. The class weight is then defined as

(26) $$w_{c}=\frac{1}{\sqrt{f_{c}}},$$

and subsequently normalized by its mean value. The square-root formulation reduces the influence of extreme weights for very rare classes while still increasing the contribution of foreground voxels.

The weighted cross-entropy term is

(27) $$L_{WCE}=-\frac{1}{N}\sum_{i=1}^{N}\sum_{c\in C}w_{c}\;t_{i,c}log\left({\hat{p}}_{i,c}+\varepsilon \right),$$

where N is the number of voxels in a mini-batch and $t_{i,c}$ is the one-hot encoded reference label, and *ε* is a small positive numerical stabilizer.

The Tversky term is introduced to optimize the overlap of sparse damage regions and to control the relative penalty assigned to false positives and false negatives [53,54]. For class c, the Tversky index is defined as

(28) $$T_{c}=\frac{{TP}_{c}+\varepsilon }{{TP}_{c}+\alpha_{c}{FP}_{c}+\beta_{c}{FN}_{c}+\varepsilon },$$

where ${TP}_{c}$, ${FP}_{c}$ and ${FN}_{c}$ are soft voxel-wise equivalents of true positives, false positives and false negatives. Since the main objective of this part of the study is damage segmentation, the Tversky component is computed for the foreground classes:

(29) $$L_{Tversky}=\frac{1}{\left|C_{fg}\right|}\sum_{c\in C_{fg}}\left(1-T_{c}\right),$$

where $C_{fg}=\{1,2\}$ denotes the foreground damage classes. The complete loss function is

(30) $$L=\lambda_{CE}L_{WCE}+\lambda_{T}L_{Tversky},$$

where $\lambda_{CE}$ and $\lambda_{T}$ determine the contribution of the voxel-wise and overlap-oriented terms. This formulation was selected because it reflects the requirements of the present XCT problem: stable learning of all voxels and increased sensitivity to sparse foreground damage.

#### 2.2.4. Patch-Based Training and Soft-Averaged Inference

The XCT volumes analyzed in this article are processed using patch-based training and inference. This is necessary because full volumetric data are large and because damage voxels are sparse. Training on randomly selected full-volume subregions enables memory-efficient optimization, while class-aware sampling increases the probability of presenting the model with patches containing delamination-like and crack-like regions.

Let $X_{m}$ denote the m-th volumetric patch and let $c_{m}$ be the target class selected during patch sampling. The sampling distribution is controlled by

(31) $$Pr\left(c_{m}=c\right)=q_{c},\;\;\sum_{c\in C}q_{c}=1,$$

where $q_{c}$ is the probability of selecting class c as the sampling target. For foreground classes, a candidate patch can be accepted only if it contains a minimum number of voxels from the selected damage class. This strategy prevents the training process from being dominated by empty or nearly empty background patches.

At inference time, the entire XCT volume is segmented by moving overlapping 3D patches through the volume. Instead of assigning final labels independently for each patch, the method accumulates soft class scores from all overlapping patch predictions. Let $M\left(i\right)$ be the set of patches covering voxel i, and let $W_{m}\left(i\right)$ be the spatial weight of voxel i in patch m. The accumulated class score is

(32) $$S_{c}\left(i\right)=\sum_{m\in M\left(i\right)}W_{m}\left(i\right)\;{\hat{p}}_{m,c}\left(i\right),$$

and the accumulated weight is

(33) $$A\left(i\right)=\sum_{m\in M\left(i\right)}W_{m}\left(i\right).$$

The soft-averaged class score is then

(34) $${\bar{p}}_{c}(i)=\frac{S_{c}(i)}{A(i)}.$$

In computational implementation, voxels with an accumulated weight below machine precision for single-precision arithmetic are assigned unit weight before division. The final raw prediction is obtained from ${\bar{p}}_{c}(i).$ This inference strategy reduces patch-boundary artifacts and improves the spatial consistency of the segmentation in the full XCT volume.

#### 2.2.5. Postprocessing and Quantitative Segmentation Metrics

The raw output of the 3D U-Net is further analyzed using conservative post-processing. The purpose of this stage is not to impose a strong priority on the damage morphology but to remove obvious isolated artifacts while preserving small real defects. This is particularly important in the present study because early fatigue damage may have a small volume and aggressive filtering could remove physically meaningful crack- or delamination-like regions.

For a connected component ${Ω}_{j}^{\left(c\right)}$ predicted as class c, its volume is defined as

(35) $$V_{j}^{\left(c\right)}=\left|{Ω}_{j}^{\left(c\right)}\right|.$$

For crack-like components, a simple elongation descriptor can also be used:

(36) $$E_{j}=\frac{a_{j,1}}{a_{j,2}},\;\;a_{j,1}\geq a_{j,2}\geq a_{j,3},$$

where $a_{j,1}$, $a_{j,2}$ and $a_{j,3}$ are the principal-axis lengths of the component. Such descriptors are used only as mild geometric constraints, so that small but plausible damage components are not removed.

In the segmentation task, the evaluation is performed in a voxel-wise manner and separately for each class using a one-vs-rest scheme. For a given class c, all voxels assigned to this class are treated as positive samples, whereas voxels belonging to the remaining classes are treated as negative samples. The corresponding voxel-level counts ${TP}_{c}$, ${FP}_{c}$, ${FN}_{c}$ and ${TN}_{c}$ are then used to calculate ${Precision}_{c}$, ${Recall}_{c}$ and ${F1}_{c}$ analogously to the classification metrics defined in Section 2.1.2, but separately for each segmentation class and at the voxel level. In addition to these metrics, two overlap-based segmentation measures are used, namely the intersection-over-union coefficient and the Dice coefficient, defined as:

(37) $${IoU}_{c}=\frac{{TP}_{c}}{{TP}_{c}+{FP}_{c}+{FN}_{c}},$$

and

(38) $${Dice}_{c}=\frac{2{TP}_{c}}{2{TP}_{c}+{FP}_{c}+{FN}_{c}}.$$

These metrics provide class-specific information on the spatial agreement between the predicted and reference voxel labels. In particular, ${IoU}_{c}$ measures the ratio between the correctly predicted region and the union of the predicted and reference regions, whereas ${Dice}_{c}$ emphasizes the overlap between both regions and is commonly used in semantic segmentation tasks. Although ${Dice}_{c}$ is algebraically equivalent to ${F1}_{c}$ when both are computed from the same voxel-level confusion counts, it is reported separately because it is a standard overlap metric in image and volume segmentation studies. In this article, these metrics are used to compare raw and post-processed segmentations and to assess segmentation quality separately for the considered damage classes. In addition to per-volume results, global metrics are computed after summing the voxel-level confusion counts over all holdout volumes. This avoids overinterpreting individual cases and provides a more representative estimate of the performance of the 3D U-net model for sparse and heterogeneous fatigue damage.

## 3. Materials and Methods

The classification of internal damage was performed on a set of XCT scans for composite specimens subjected to fatigue loading. The description of materials, observed damage, and parameters of acquisition of XCT scans and their initial preprocessing are provided in the following section.

### 3.1. Materials and Thermomechanical Fatigue Degradation

The glass fiber-reinforced polymer (GFRP) composite specimens considered in this study were manufactured and supplied by Izo-Erg S.A. (Gliwice, Poland). They were composed of epoxy resin and 14 layers of plain weave E-glass fabric with a weight of 200 g/m^2^ used as reinforcement. The manufacturing was performed using the hot press technique (for more details on the manufacturing process and mechanical properties, see [28,55]). The manufactured sheets with a thickness of 2.5 mm were cut to specific dimensions of the specimens of 10 mm × 100 mm using a circular saw.

**Figure 1:**
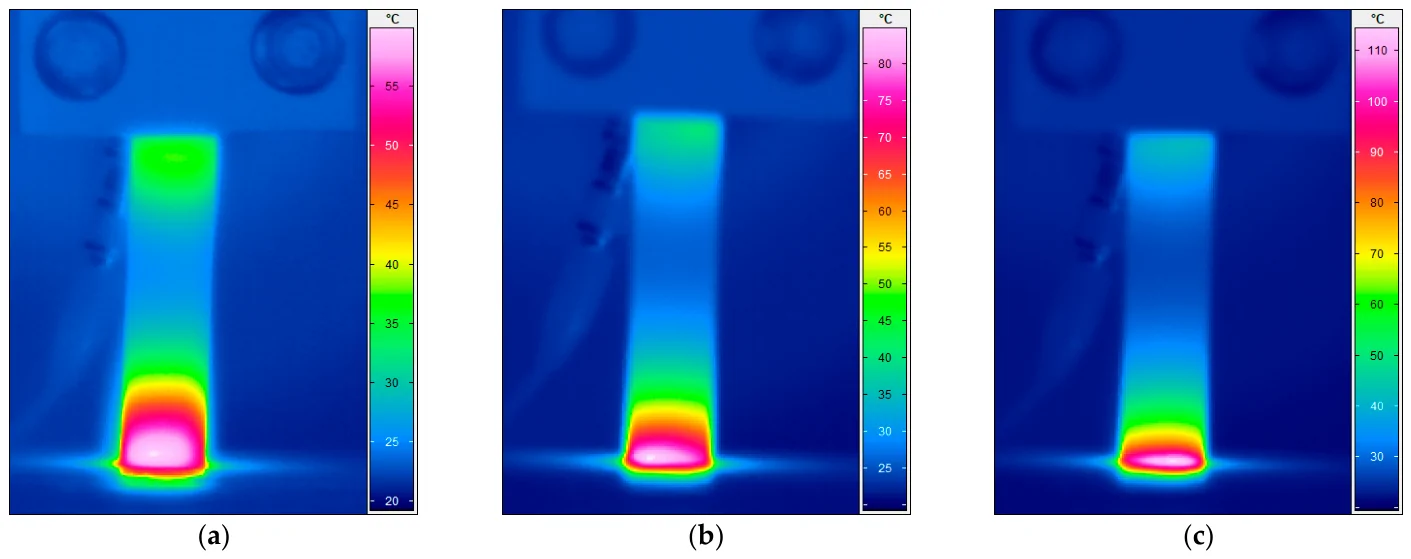
The exemplary thermograms of the considered specimens demonstrating the self-heating effect at various stages: (**a**) at 60 °C, (**b**) at 85 °C, (**c**) at 115 °C.

The specimens were then subjected to cyclic loading in the custom-designed fatigue test rig. Each specimen was clamped at one end, with an effective free length of 50 mm, and excited at the opposite end by the TIRA^®^ TV-51120 electrodynamic shaker (TIRA, Schalkau, Germany) through a force sensor. The fully reversed (R=−1) bending loading was applied with an initial excitation force of 90 N, which is equivalent to the initial stress of ca. 432 MPa (ca. 68% of the flexural strength of the tested composite—cf. [55]), and a frequency of 30 Hz. This implies the development of the self-heating effect in tested specimens resulting from the hysteretic behavior of a viscoelastic polymer matrix of a tested composite and, consequently, a temperature increase in the locations of stress concentrations. The frequency of 30 Hz was selected based on our previous thermomechanical fatigue studies [56], in which this frequency provided a stable and sufficiently pronounced self-heating response for the investigated composite. The vibration response was monitored using the Polytec^®^ PDV-100 laser Doppler vibrometer (Polytec, Waldbronn, Germany), while the surface temperature distribution was recorded using the InfraTec VarioCAM hr infrared camera (InfraTec, Dresden, Germany) focused on the specimens’ surface. More details about the experimental setup can be found in [28]. To observe various stages of structural degradation due to fatigue, the loading was applied until a specific self-heating temperature was reached on the surface of a tested specimen, after which the loading was interrupted. The range of considered self-heating temperatures was initially set at 35–115 °C with a step of 5 °C. The selection of this range was also considered in relation to the thermal characteristics of the EP GC 201 material. Previous dynamic mechanical analysis of the tested material showed that the glass-transition temperature depends on the heating rate and excitation frequency, with values of approximately 124–157 °C reported for the investigated conditions and a reference value of 134.36 °C [55]. The final temperature range was based on the observed structural degradation. After analysis (see [28]), the lower limit was raised to 60 °C, since no internal damage was detected below this temperature, while the upper limit of 115 °C was retained due to frequent loss of structural integrity above it. Thus, 115 °C is not a predefined degradation threshold, but a conservative experimental limit supported by the thermal characteristics of the tested material. The exemplary thermograms that present the self-heating effect in the tested specimens are shown in Figure 1. As can be observed, the temperature distribution changes with the development of damage due to changes in stress distribution in a loaded specimen.

**Figure 2:**
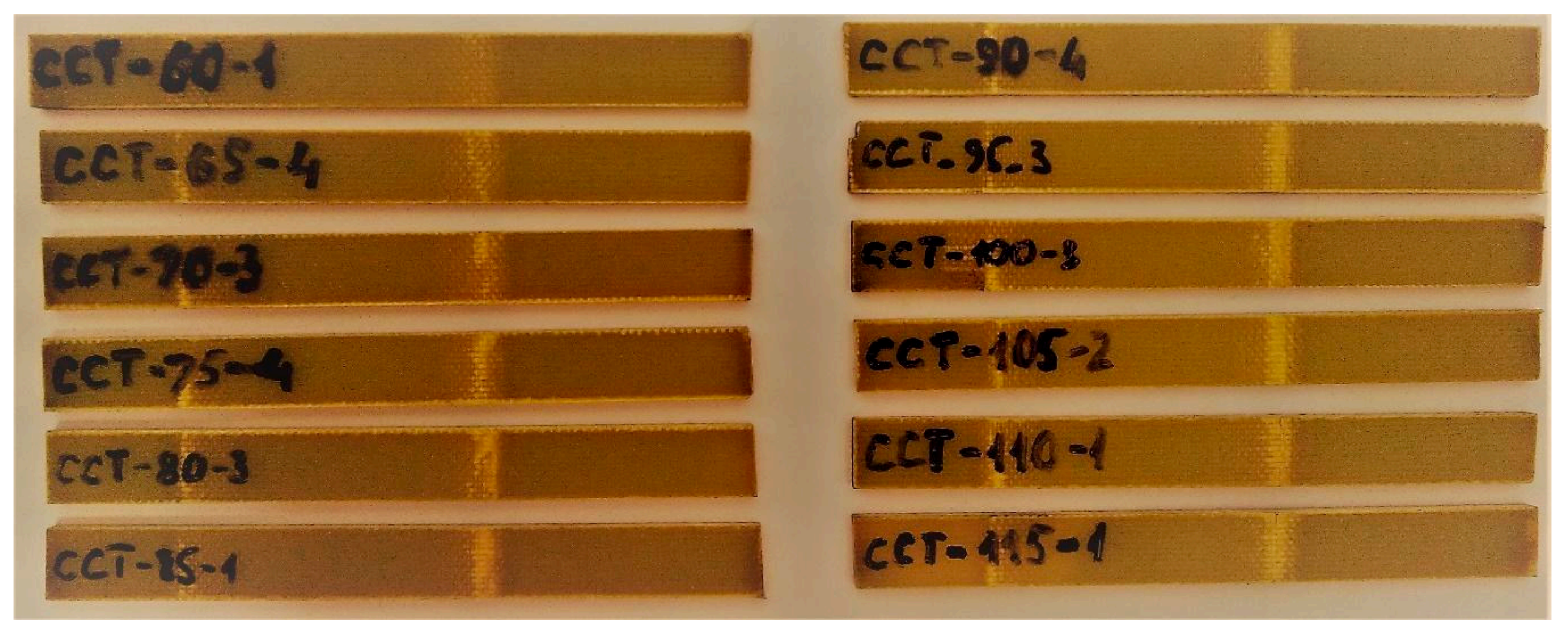
The set of tested specimens presenting the evolution of structural degradation due to fatigue with an increase in the self-heating temperature.

For each self-heating temperature value considered in this study, four specimens were tested, resulting in 48 tested specimens in total. The evolution of degradation for specific self-heating temperature values is presented in Figure 2. Here and further, the labeling of specimens is assumed as follows: CCT-{self-heating temperature}-{number of a specimen within a group}.

In Figure 2, it can be clearly seen that fatigue damage is manifested by lighter regions on the specimens’ surface, with increasing intensity and area with increased self-heating temperature. Lighter regions represent delamination, while in some cases surface cracks are also visible.

### 3.2. X-Ray Computed Tomography Scanning and Initial Image Processing

To evaluate the internal structure of the considered specimens, including damage resulting from fatigue testing, XCT testing was performed. The main advantage of this NDE technique is the possibility of evaluating internal damage in three spatial dimensions, which significantly simplifies the analysis of the resulting XCT scans and their application as an input in ML algorithms.

**Figure 3:**
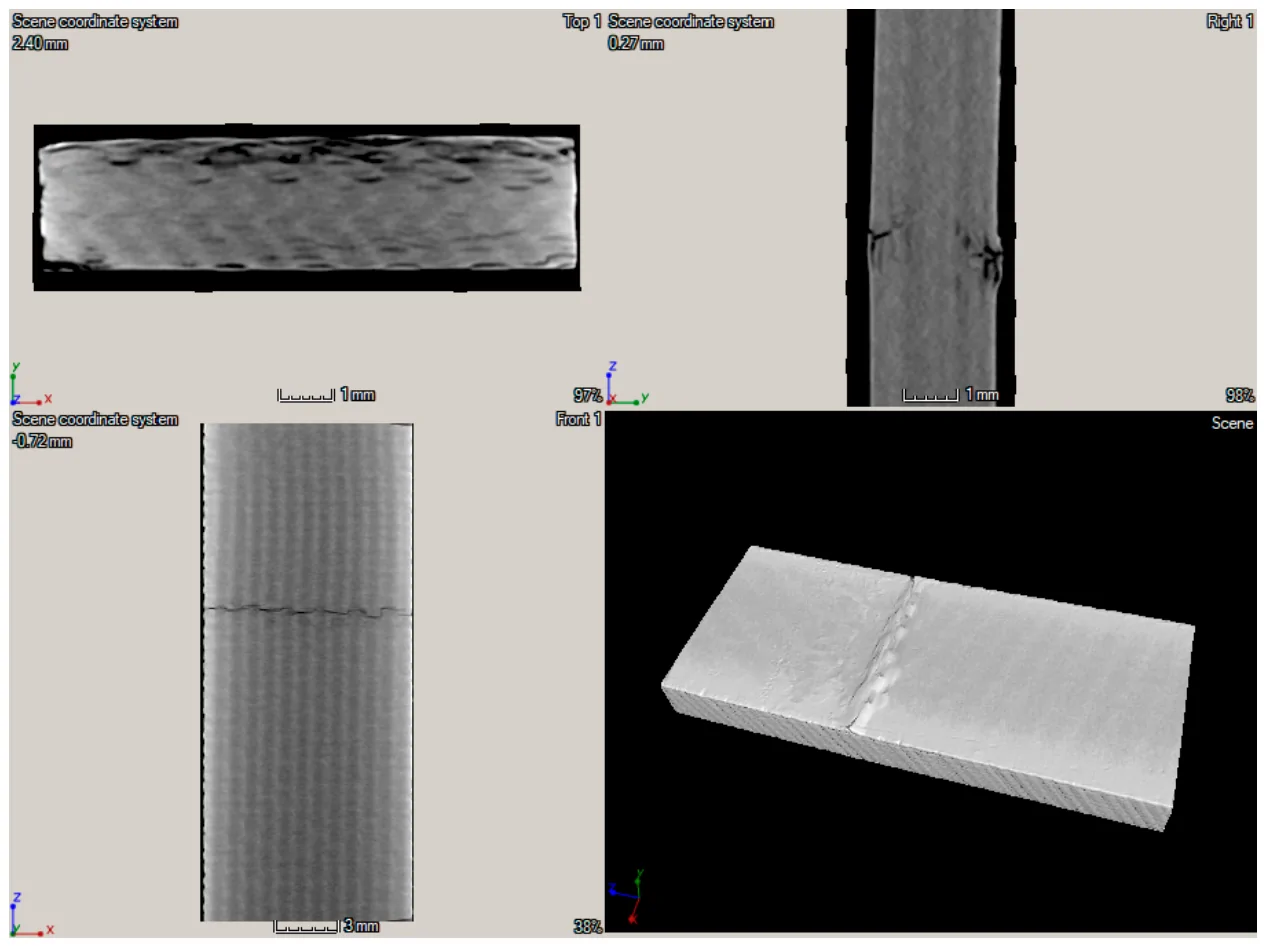
An example of an XCT scan for the CCT-115-1 specimen.

XCT testing was performed using the Phoenix v|tome|x m 300 XCT scanner manufactured by GE^®^ Measurement and Control Solution (Billerica, MA, USA) with a maximal power of 500 W at 300 kV voltage and the GE^®^ detector of DXR type with 400 × 400 mm active area. After preliminary inspection of the considered specimens, it was found that the extent of damage in the longitudinal direction of the specimen does not exceed 10 mm in all considered cases. Accordingly, the region of interest for each specimen is limited to a volume of 10 × 10 × 2.5 mm to achieve the best possible resolution of the XCT scans. The dimensions of a single voxel were 0.025 × 0.025 × 0.025 mm, which revealed a good representation of internal damage in the tested specimens. The obtained XCT scans were imported into the tomograph-dedicated software Volume Graphics VGStudio MAX 2024.4 (Heidelberg, Germany) to improve contrast between damaged and healthy regions. For the annotation of post-processed images, Fiji v2.9.0 software was used [57]. An example of an XCT scan of the tested structure is presented in Figure 3.

The improved XCT scans were then exported from VGStudio MAX in the form of 2D slices. Some slices from the upper and lower areas of the specimens were excluded from consideration due to incompleteness of the slice caused by the deformation of the specimen during fatigue testing and the loss of its initial flatness. According to this, the average number of slices for each considered specimen was 89, while the total number of 2D slices for all considered specimens was 4284. In the form of graphical files, 2D slices were imported into MATLAB^®^ R2025b (MathWorks, Natick, MA, USA) used for initial processing of the resulting images.

**Figure 4:**
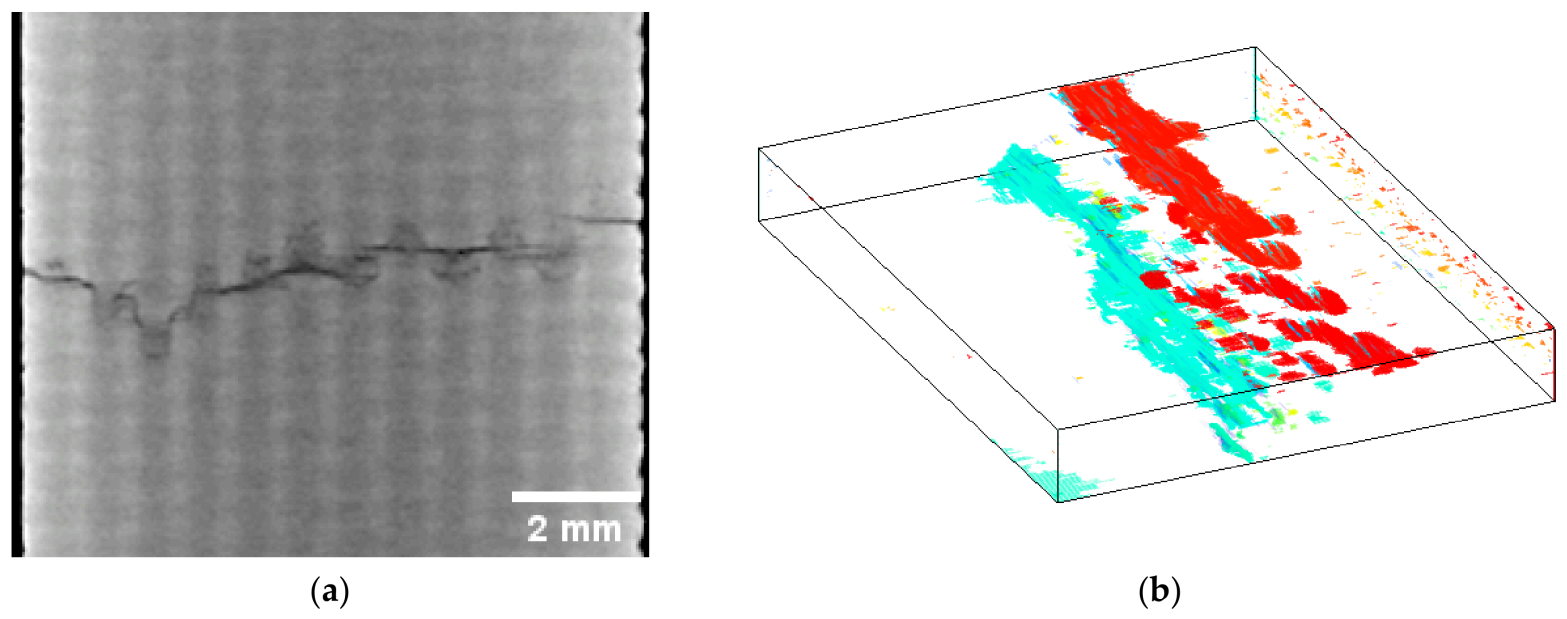
Initial processing of XCT scans: (**a**) 2D slice of the tomogram with visible texture and illumination inhomogeneity; (**b**) labeled 3D damage features extracted from a representative tomogram.

The processing of 2D slices of XCT scans aimed at the extraction of characteristic damage types, namely delamination and cracks, for further extraction and evaluation of characteristic features of particular types of damage, as well as for preparation of input data for ML. The initial processing algorithm was based on two steps. In the first step, texture-based image segmentation was performed, which consisted of adaptive thresholding and filtering to reduce noise and enhance distinguishability between healthy and damaged areas of the specimens (see Figure 4a). Additionally, morphological operations were applied to limit the detected objects to the mentioned types of damage. During the second step, the identification of delamination and cracks was performed based on geometric relationships between these types of damage. After processing, the 2D slices were reconstructed back to 3D arrays for further processing and visualization purposes. The detailed description of the processing algorithm is presented in [28]. The extracted 3D features were labeled to distinguish them and prepare them for further classification procedures. The resulting labeled features are visualized in Figure 4b, where the colors denote individual connected components.

In Figure 4, it can be observed that the extracted set of 3D damage features contains both structural damage and artifacts, especially near the specimen surfaces and corners. These artifacts are the result of XCT scanning and subsequent preprocessing steps. Since such objects are commonly present in CT-based damage extraction, they were retained at this stage and treated as separate connected components in the subsequent object-level analysis. In the present study, the analysis was extended from a single representative tomogram to a complete dataset consisting of 53 three-dimensional damage masks. Consequently, the number of analyzed components was substantially greater than in the initial representative case.

**Figure 5:**
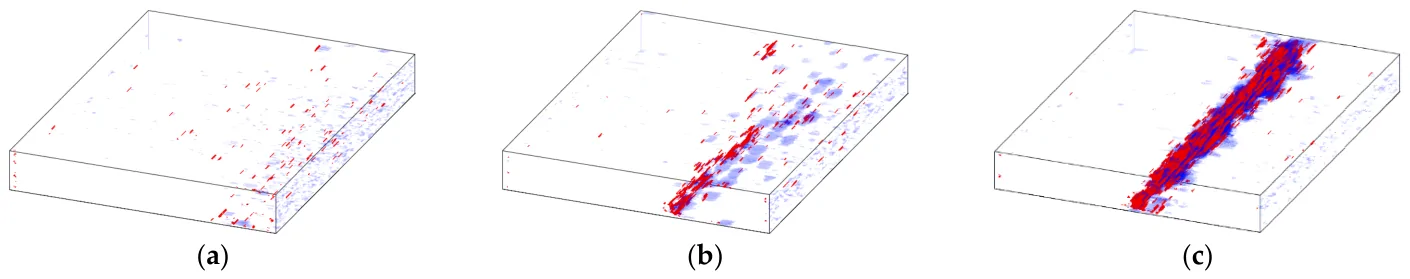
The exemplary reconstructions of XCT scans after initial processing for the maximal self-heating temperature registered during thermomechanical fatigue of: (**a**) at 60 °C, (**b**) at 85 °C, (**c**) at 115 °C. The colors denote the type of damage: red—cracks, blue—delamination.

The exemplary XCT scans after initial processing with indication of predefined types of damage are presented in Figure 5.

### 3.3. Classification and Segmentation Algorithms

#### 3.3.1. Morphological- and Intensity-Feature-Based Classification

Following the XCT preprocessing described in Section 3.2, the extracted three-dimensional connected components were used as object-level observations for supervised classification. Each component was assigned to one of two damage classes: D1, corresponding to delamination, and D2, corresponding to crack. Since the crack class was less frequent than the delamination class, D2 was treated as a positive class in the ROC-AUC and PR-AUC analyses. The classification procedure described in this section was performed on tabular feature vectors extracted from previously identified 3D components. Therefore, it should be distinguished from the voxel-wise segmentation procedure described in Section 3.3.2.

The final classification dataset was prepared from 53 CCT cases. The cases were divided into a training subset and an independent holdout subset at the level of complete CCT cases, so that components originating from the same case were not simultaneously present in both subsets. This strategy was used to eliminate information leakage between training and testing data. Only components with valid correspondence to the raw XCT volume and valid intensity information were retained. Components without reliable intensity values or with fewer than 10 voxels in the local-background region were excluded from the final dataset. The local background was defined separately for each component as the non-component neighborhood surrounding that component in the XCT volume. Components with too few local-background voxels were excluded because local contrast, local-background variability, and local CNR could not be estimated reliably. No separate artifact-related, mixed, or uncertain class was introduced in the component-level classification dataset. Components were retained for classification only when the initial image processing workflow assigned them to one of the two considered damage classes, D1 or D2, and when reliable feature values could be calculated. Components that could not be reliably matched to the raw XCT volume or for which local intensity-based features could not be calculated were excluded. Nevertheless, possible residual label uncertainty caused by artifact-related or mixed connected components is acknowledged as a limitation of the component-level reference-labeling procedure.

For each retained component, features were calculated from two complementary sources of information: the binary 3D component mask and the corresponding grayscale XCT volume. The first group included morphological features derived from the three-dimensional geometry of the connected component. These features included component volume, surface area, three principal-axis lengths, three-dimensional solidity, circumferential diameter, inscribed-sphere diameter and sphericity-related features, namely WadellSphericity, TickellSphericity, RilleySphericity, CoxBudhuSphericity and TrueSphericity. Such features describe the size, compactness, spatial extent, local thickness, and shape regularity of the component. These features were selected to observe differences in the morphological geometry of the cracks and delamination components.

The second group included XCT intensity-based and statistical descriptors calculated from the raw XCT voxel intensities assigned to the component and from its local background. These features included mean and median component intensity, fifth and ninety-fifth intensity percentiles, local contrast, intensity standard deviation, interquartile range, skewness, kurtosis, and local-background intensity standard deviation and local contrast-to-noise ratio.

Three feature sets were prepared for comparative analysis. The first set, denoted as INT, contained 11 XCT intensity-based and statistical features. The second set, denoted MORPH3D, contained 13 three-dimensional morphological features independent of component position and orientation. The third set, denoted FUSED, combined the INT and MORPH3D feature groups and consisted of 24 features.

Before model training, source identifiers, object identifiers, and class labels were excluded from the input feature matrix. The response variable was the component class. Features containing NaN or Inf values in the training or holdout subsets were removed because such values may result from undefined or unreliable feature calculations for degenerate components, for example, when local-background information is insufficient or when derived intensity measures involve division by very small or zero values. Features with zero standard deviation in the training subset were also excluded because they were constant, provided no discriminative information for classification, and could lead to undefined values during z-score standardization. No imputation of missing values was applied. Standardization using z-score scaling was applied to classifiers sensitive to feature scaling, namely RBF-SVM, k-nearest neighbors and the feed-forward neural network. Tree-based classifiers were trained on the original feature values.

Feature selection was based on the minimum redundancy maximum relevance criterion (mRMR) [49], whereas its fold-wise application inside the grouped cross-validation procedure is described in the following section together with hyperparameter tuning.

Five families of classifiers were evaluated for each feature representation. The first model was a shallow decision tree implemented using *fitctree*. This model was used as an interpretable rule-based classifier. The maximum number of splits was limited to preserve interpretability and depended on the feature representation. Surrogate splits were disabled. The second model family consisted of bagged decision trees implemented using *fitcensemble* with bootstrap aggregation and decision tree learners. In this case, an ensemble of 100 trees was used to improve classification stability with respect to a single decision tree. The third classifier was a support vector machine with a radial basis function kernel (RBF-SVM), implemented using *fitcsvm*. The RBF-SVM was selected as a non-linear margin-based classifier suitable for separating the two damage classes in multidimensional feature space. Input features were standardized before fitting this model. The tuned RBF-SVM parameters were the regularization parameter, BoxConstraint, and the kernel-scale parameter, KernelScale, both selected separately for each feature representation. The fourth classifier was k-nearest neighbors, implemented using *fitcknn*. This model represented a distance-based classification approach in the standardized feature space. Its tuned parameter was the number of neighbors, selected separately for each feature representation. The fifth classifier was a shallow feed-forward neural network implemented using *fitcnet*. The tuned neural network parameter was the number of hidden neurons, whereas the iteration limit was fixed during model selection. The neural network was trained on the same tabular feature vectors as the remaining classifiers. It consisted of one hidden layer, with its size adjusted to the dimensionality of the input feature set. The feed-forward neural network was therefore used only as an object-level classifier of extracted component features and not as an image segmentation model. It did not replace the 3D U-Net segmentation approach.

Model selection and hyperparameter tuning were performed separately for each feature representation, namely INT, MORPH3D, and FUSED. For each classifier family, a predefined grid of hyperparameter values was evaluated using grouped 10-fold cross-validation on the training subset. For each candidate configuration, mRMR feature ranking, selection of the retained features, model fitting, and, for scale-sensitive classifiers, standardization were repeated independently within each grouped cross-validation fold using only the fold-training data. The corresponding validation fold was used only to obtain predictions and calculate cross-validation performance. The grouping was performed at the CCT-case level. This means that all components extracted from one CCT case were assigned to the same cross-validation fold. Such a procedure was used because components originating from the same XCT case may share acquisition conditions, preprocessing characteristics, and specimen-specific damage morphology. Without grouping, components from the same CCT case could appear simultaneously in the training and validation folds, which could lead to overly optimistic validation results.

A predefined, limited hyperparameter grid was used to compare classifier configurations in a controlled and reproducible way. For the shallow decision tree, the maximum number of splits was selected from {8, 16, 32, 64}. For bagged decision trees, the maximum number of splits was selected from {32, 128}, while the number of learning cycles was fixed at 100. For the RBF-SVM classifier, the BoxConstraint parameter was selected from {1, 10} and the KernelScale parameter from {1, 10}. For the k-nearest neighbors classifier, the number of neighbors was selected from {3, 7, 15}. For the feed-forward neural network, the hidden layer size was selected from {10, 30}, while the iteration limit was set to 400. Therefore, the selected configuration should be interpreted as the best-performing configuration within the predefined grid, not as a continuous global optimum of the hyperparameters.

In addition to model-specific hyperparameters, the number of retained features was also treated as a hyperparameter. Feature ranking was performed using the minimum redundancy maximum relevance criterion. The mRMR ranking was recomputed independently inside each cross-validation fold using only the training part of that fold. Then, the first ranked features were selected, with the number of retained features chosen from {3, 5, 8, 10, all available features}; here, all available features corresponded to 11 features for INT, 13 for MORPH3D, and 24 for FUSED. This feature-selection procedure was applied separately for the INT, MORPH3D, and FUSED feature representations. It ensured that feature selection was included inside the cross-validation loop and did not use information from the validation data.

For each candidate configuration, predictions from all validation folds were pooled and used to calculate cross-validated performance metrics. Balanced accuracy, defined as the arithmetic mean of class-wise recalls, was used as the primary model selection criterion because the classification problem was imbalanced and class D2, corresponding to crack damage, was less frequent than class D1. If two configurations obtained similar balanced accuracy, the following criteria were used successively: F1-score for class D2, macro-F1, cross-validation error, and the smaller number of selected features.

After selecting the best-performing configuration within the predefined grid, the final classifier was retrained using the complete training subset and the corresponding selected feature set. The independent holdout subset was not used at any stage of hyperparameter tuning, feature selection, or model selection. It was reserved exclusively for the final assessment of the selected models. The final holdout evaluation included overall accuracy, precision for D2, recall for D2, F1-score for D2, ROC-AUC and PR-AUC. Confusion matrices were calculated for all model and feature-set combinations to analyze the dominant types of misclassification. In addition, the resubstitution error was calculated only for diagnostic purposes, in order to compare training-set fit with cross-validation performance and assess possible overfitting.

#### 3.3.2. 3D UNet-Based Segmentation

The voxel-wise segmentation of XCT volumes was implemented according to the volumetric semantic segmentation formulation introduced in Section 2.2. During training, the input tensor in Equation (13) was represented by a grayscale volumetric patch $X\in R^{32\times 32\times 32\times 1}$, and the network estimated the dense label map $\hat{Y}$ defined in Equation (14). For each voxel i, the model produced the score vector ${\hat{p}}_{i}$ and class scores ${\hat{p}}_{i,c}$ defined in Equations (15) and (16). The raw voxel label ${\hat{y}}_{i}^{raw}$ was assigned according to Equation (17), and class-dependent acceptance thresholds $\tau_{c}$ were then applied according to Equation (18). The threshold values were $\tau_{0}=0.00$, $\tau_{1}=0.20$ and $\tau_{2}=0.35$.

Before training, each raw XCT volume was paired with the corresponding three-dimensional damage mask obtained from the initial image-processing workflow described in Section 3.2. The raw volume X and the reference label volume were spatially matched, cropped, normalized, and stored as prepared image-label pairs. Cropping was used to remove boundary artifacts. The crop margins were [20, 20] pixels in the vertical direction, [25, 25] pixels in the horizontal direction, and [0, 0] pixels in the through-thickness direction. Grayscale XCT values were normalized using a robust procedure based on the first and ninety-ninth intensity percentiles, clipping to the normalized interval and subsequent z-score standardization. This reduced the influence of extreme intensity values. The label volumes were then remapped to the multiclass representation C={D0,D1,D2}.

The segmentation model followed the 3D U-Net formulation given in Section 2.2.2. The convolutional block $F_{l}$, encoder representation $E_{l}$, downsampled tensor $X_{l+1}$, skip-connection representation $S_{l}$, logits $z_{i,c}$ and softmax-normalized scores ${\hat{p}}_{i,c}$ corresponded to Equations (19)–(24). The network input size was 32×32×32×1. The encoder depth was $L_{enc}=4$, which was compatible with the patch size 32×32×32 and enabled memory-efficient volumetric processing.

The network was trained on volumetric patches $X_{m}$ rather than on complete XCT volumes. For each patch, the target sampling class $c_{m}$ was drawn according to Equation (31). The class-sampling probabilities $q_{c}$ were $q_{0}=0.20$, $q_{1}=0.35$ and $q_{2}=0.45$ for D0, D1 and D2, respectively. Therefore, the sparse foreground classes were sampled more frequently than would result from their natural voxel frequencies. A candidate foreground patch was accepted only if it contained a minimum number of voxels from the selected target class $c_{m}$. This minimum number was 60 voxels for D1 and 40 voxels for D2, whereas no foreground-voxel requirement was imposed for background patches. The maximum number of sampling attempts was 100. In each epoch, 800 regular training patches and 160 validation patches were sampled.

Expert-labeled two-dimensional slices were also incorporated into the training procedure. Expert annotations were prepared in Image Labeler (MATLAB) and exported as ground-truth data. The training expert dataset comprised 22 manually annotated two-dimensional XCT slices originating from 22 different non-holdout CCT cases. These expert masks were inserted into the corresponding three-dimensional label volumes at their respective slice positions, replacing the algorithmic labels at these locations. To increase their contribution during optimization, additional 3D patches were sampled with a fixed slice position so that each such patch passed through an expert-labeled slice. Each expert-labeled training slice was oversampled six times per epoch, resulting in 132 additional expert-guided 3D patches in addition to the 800 regularly sampled training patches. This preserved the volumetric context of the 3D U-Net input while increasing the contribution of the D1 and D2 expert-corrected regions during training.

A separate expert-labeled dataset was reserved exclusively for independent holdout evaluation. It comprised 10 manually annotated two-dimensional XCT slices from five holdout cases: CCT-70-1, CCT-80-4, CCT-90-3, CCT-100-2 and CCT-110-1. None of these expert annotations were used for network training or validation. All 10 holdout slices contained expert-identified crack-like damage (D2), while nine also contained delamination-like damage (D1). These annotations, therefore, provided an independent reference for assessing the agreement of the 3D U-Net predictions with expert interpretation, separately from the full-volume evaluation based on algorithmically generated 3D masks.

Domain-specific augmentation was applied only to training patches. The augmentation included random flips in the vertical, horizontal, and through-thickness directions with probabilities 0.50, 0.50 and 0.10, respectively. Random 90° rotations in the XY plane were applied with probability 0.50. Intensity-related augmentation included random intensity scaling and offset with probability 0.80, gamma transformation with probability 0.50, Gaussian noise with probability 0.50, speckle-like noise with probability 0.25, smooth bias-field variation with probability 0.35, anisotropic blurring with probability 0.25, stripe-like artifacts with probability 0.25 and random intensity clipping with probability 0.30. These transformations were selected to imitate typical XCT variability, including contrast changes, local intensity inhomogeneity, noise, and acquisition-related artifacts.

The training objective followed the class-imbalance formulation from Section 2.2.3. The empirical frequency $f_{c}$ and class weight $w_{c}$ were computed from the training voxel counts $n_{c}$ according to Equations (25) and (26). After the initial calculation, the weights were normalized by their mean value, limited to the interval [0.10, 20], and converted to single precision. In the multiclass setting, the estimated weights were additionally multiplied by [1.0, 1.0, 1.7] and normalized again by the mean value, increasing the contribution of the sparse D2 class. The final objective combined the weighted cross-entropy term $L_{WCE}$ from Equation (15) and the foreground-oriented Tversky loss $L_{Tversky}$ from Equations (28) and (29). Complete loss L followed Equation (30), with $\lambda_{CE}=0.4$ and $\lambda_{T}=0.6$. The Tversky component was computed only for $C_{fg}=\{D1,D2\}$. For D1, the Tversky parameters were $\alpha_{1}=0.50$ and $\beta_{1}=0.50$, whereas for D2 they were $\alpha_{2}=0.75$ and $\beta_{2}=0.25$. This setting increased the penalty for false-positive D2 responses while retaining sensitivity to sparse foreground damage.

The network was trained using the Adam optimizer with an initial learning rate of 10^−4^. The maximum number of epochs was 500, and the mini-batch size was 256. Training and validation volumes were loaded into RAM before optimization, and voxel indices for each class c∈C were precomputed to accelerate class-aware patch sampling. Mini-batches were prepared asynchronously using background dispatching. Validation was performed once per epoch. The network output was treated as the softmax-normalized score ${\hat{p}}_{i,c}$ from Equation (24), and the custom loss L from Equation (30) was calculated directly from these scores.

During inference, complete XCT volumes were segmented using a sliding-window patch-based procedure. The inference patch size was 32×32×32 voxels, with an overlap of 16 voxels in the first two spatial directions and 8 voxels in the through-thickness direction. For each voxel i, the set of overlapping patches M(i) was used to accumulate soft class scores. The accumulated score $S_{c}(i)$, accumulated weight A(i) and soft-averaged score ${\bar{p}}_{c}(i)$ were computed according to Equations (32)–(34). The spatial weights $W_{m}(i)$ were implemented using a raised-cosine blending window. This reduced patch-boundary artifacts and improved the spatial consistency of the predicted label map $\hat{Y}$. After soft-averaged inference, the threshold vector τ=[0.00,0.20,0.35] was applied according to Equation (18). Thus, no additional acceptance threshold was imposed on the background class, while D1 and especially D2 required a minimum softmax score to be accepted as foreground predictions.

The raw segmentation maps were further processed using the component-level postprocessing formulation from Section 2.2.5. For a connected component ${Ω}_{j}^{\left(c\right)}$ predicted as class c, the component volume $V_{j}^{\left(c\right)}$ was calculated according to Equation (23). For D2 components, the elongation descriptor $E_{j}$ from Equation (36) was additionally computed from the principal-axis lengths $a_{j,1}$, $a_{j,2}$ and $a_{j,3}$. The minimum accepted component volumes were $V_{j}^{\left(1\right)}\geq 16$ voxels for D1 and $V_{j}^{\left(2\right)}\geq 10$ voxels for D2. For D2, the minimum elongation threshold was $E_{j}\geq 1.3$. These thresholds were intentionally mild to avoid removing small but physically plausible fatigue-damage regions.

An additional artifact-suppression rule was applied to D1 components located in the upper-right corner region of the selected slices. This correction was performed on connected components ${Ω}_{j}^{\left(1\right)}$, not on the entire region of interest. The predefined corner region corresponded to the upper 45% and right 45% of the image area. A D1 component was removed only if at least 70% of its volume was located in this corner region and if its volume exceeded 100 voxels. The D1 components located near D2 were protected using a dilation-based neighborhood criterion with radius 6. This conservative rule was introduced to suppress systematic corner artifacts while preserving plausible D1 regions spatially related to D2.

## 4. Results and Discussion

### 4.1. Experimental Dataset Organization and Evaluation Strategy

The experimental evaluation was organized at the level of complete CCT cases to ensure a consistent assessment and minimize the risk of information leakage for both tasks considered in this study. The same independent holdout subset was used for the component-level classification task introduced in Section 2.1 and implemented in Section 3.3.1, as well as for the voxel-wise segmentation task introduced in Section 2.2 and implemented in Section 3.3.2. Six complete CCT cases were withheld from all training, validation, feature-selection and hyperparameter-tuning procedures: CCT-110-1, CCT-100-2, CCT-90-3, CCT-80-4, CCT-70-1 and CCT-60-2. These cases were used only for the final independent evaluation. Such a case-level split was necessary because components and voxels originating from the same XCT scan share acquisition conditions, preprocessing characteristics, and specimen-specific damage morphology. Therefore, separating data at the component or voxel level could lead to information leakage and overly optimistic performance estimates.

For the classification task, the remaining non-holdout CCT cases were used to construct the training dataset of three-dimensional damage components extracted according to the preprocessing workflow described in Section 3.2. Each retained component was represented by the intensity-based, morphological, or fused feature sets described in Section 3.3.1. Model selection, feature selection, and hyperparameter tuning were performed only within the training subset using grouped cross-validation at the CCT-case level. This means that all components extracted from a given CCT case were assigned to the same cross-validation fold. The independent holdout cases were not used during mRMR ranking, feature selection, scaling, model tuning, or classifier selection. They were reserved exclusively for the final assessment of the classification performance reported in Section 4.3.

For the segmentation task, the same holdout CCT cases were also excluded from network training and validation. The remaining non-holdout volumes were divided into training and validation subsets for the 3D U-Net procedure described in Section 3.3.2. In contrast to the classification task, where observations were individual connected components, the segmentation task operated on volumetric image-label pairs and on three-dimensional patches sampled from these volumes. Training patches were generated from training volumes using class-aware sampling to increase the representation of sparse foreground damage classes. Validation patches were sampled from the validation volumes and used only to monitor the training process. When expert-labeled two-dimensional slices were available for non-holdout cases, the corresponding CCT cases were kept in the training subset so that expert-corrected annotations contributed only to model learning and not to validation. The final segmentation performance was then evaluated on the independent holdout cases using both algorithmic three-dimensional reference labels and expert-labeled two-dimensional holdout slices, as reported in Section 4.4 and Section 4.5.

### 4.2. Initial Preprocessing and Feature Selection

The initial object-level data included 17,312 extracted three-dimensional components. After removing objects with invalid intensity information, 12,757 components were retained for the final feature-based classification analysis. The retained dataset included components extracted from 53 CCT cases. These cases were divided into 47 CCT cases used for model training, grouped cross-validation, feature selection and hyperparameter tuning, and 6 independent CCT cases reserved for final holdout evaluation. The split was performed at the level of complete CCT cases, taking into account the same distribution of degree of degradation, represented by the volume of cracks and delaminations, in training and final holdout tests.

The final dataset was imbalanced with respect to the two considered damage classes (see Table 1). Class D1, which corresponds to delamination, was represented by 10,395 components, while class D2, which corresponds to crack-like damage, was represented by 2362 components. In the training subset, 9014 delamination components and 2135 crack components were available. The independent holdout subset contained 1381 delamination components and 227 crack components. Since the crack class was less frequent, particular attention was paid to the class-specific metrics for D2 during the subsequent classifier evaluation.

For each retained component, features were extracted from two complementary sources of information: the three-dimensional binary component mask and the corresponding grayscale XCT intensity data. Morphological features were calculated from 3D components, while intensity-based features were calculated from raw XCT voxel values belonging to the component and its local background.

Three subsets of features were prepared for comparative analysis and described in Table 2. The INT feature set contained 11 features related to intensity distribution and local contrast. The MORPH3D feature set contained 13 three-dimensional morphological features computed in physical units after the voxel-size calibration and independent of component position, orientation, and voxel-grid scale [47]. The FUSED feature set combined both groups and consisted of 24 features. This division made it possible to assess whether the delamination and crack components could be separated using intensity-based features alone, morphological features alone, or the combined FUSED representation.

The pre-processing of the tabular dataset included removal of non-predictive identifiers and components for which reliable intensity-based descriptors could not be calculated. Components were excluded when their binary mask could not be matched unambiguously with the corresponding raw XCT grayscale volume, when no valid intensity values were available, or when the local-background region contained fewer than 10 voxels. Predictors containing NaN or Inf values in the training or holdout subsets, as well as predictors with zero standard deviation in the training subset, were removed before feature ranking and model training. This filtering was not specific to mRMR, but was required to avoid undefined numerical operations during scaling, feature selection, and classifier fitting. No imputation procedure was applied.

Feature redundancy was also assessed before classification. No exact or affine duplicate features were found in the final feature sets. Several strongly correlated feature pairs were identified; therefore, redundancy was not addressed by a global correlation-based exclusion step. Instead, redundancy was controlled during model selection by applying the minimum redundancy maximum relevance criterion within the cross-validation procedure. mRMR-based selection was performed only on the training part of each fold, preventing information leakage from validation data into feature selection.

The mRMR ranking calculated for the FUSED representation showed that the most informative features were not limited to a single feature group. The highest-ranked feature was Wadell sphericity, followed by the inscribed-sphere diameter and local contrast. This indicates that the separation between the delamination and crack components was governed by both geometrical compactness and local grayscale visibility. The intensity kurtosis and the local contrast-to-noise ratio were also highly ranked, showing that the shape of the intensity distribution and the contrast relative to the local background contributed to the damage-class separation.

The feature selection results confirm that the classification problem cannot be reduced to a simple size-based separation of components. The highest-ranked features, including sphericity-related morphology, component thickness, local contrast, and higher-order intensity statistics, are presented in Table 3. This suggests that the delamination and crack components differ simultaneously in three-dimensional morphology and local grayscale-intensity characteristics. Therefore, the FUSED representation was expected to provide the most complete input for classification, as it combined morphological and intensity-based information in a single feature vector.

### 4.3. Classification Results

The classification performance was evaluated on the independent holdout subset for all combinations of feature representation and five classifier families described in Section 3.3.1.

**Figure 6:**
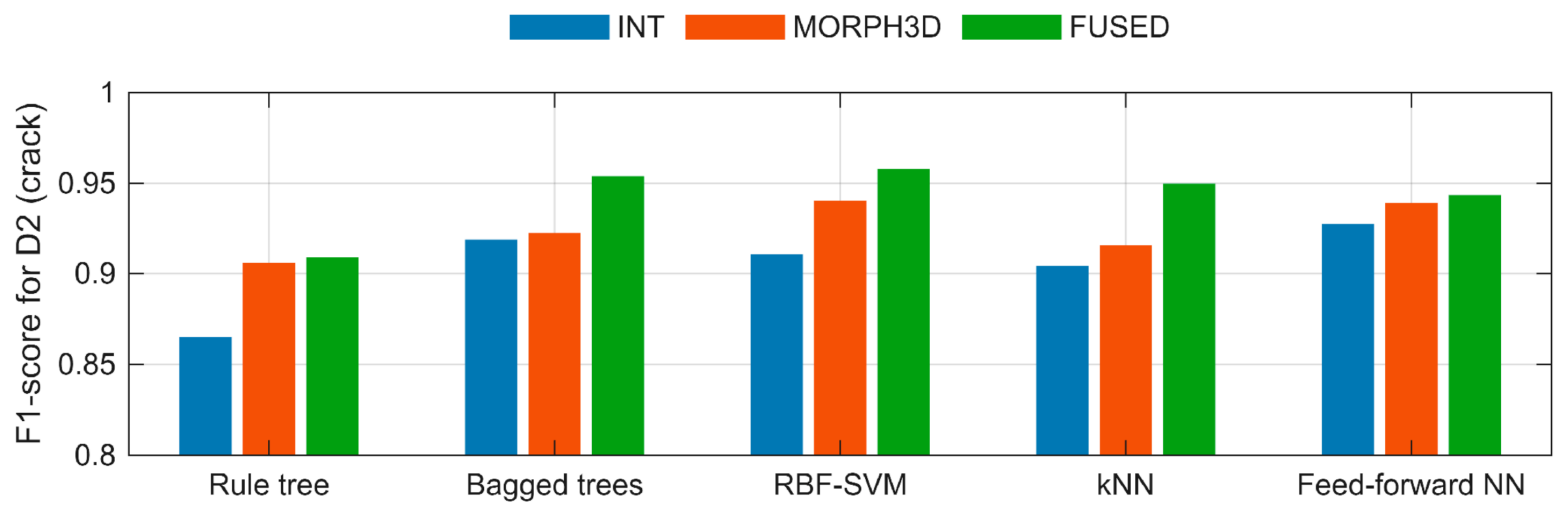
Comparison of classification performance for the INT, MORPH3D and FUSED feature representations. The F1-score is reported for the minority D2 class, corresponding to crack damage.

Since the crack class D2 was less frequent than the delamination class D1, the interpretation of the results was not based solely on overall accuracy. Particular attention was paid to precision, recall, and F1-score for class D2. In addition, ROC-AUC and PR-AUC were reported to evaluate the ranking ability of classifiers and their performance under class-imbalanced conditions. The comparison of the F1-score values for class D2 is shown in Figure 6. This metric was selected for graphical presentation because it directly reflects the quality of classification of the minority crack class.

As shown in Figure 6, the INT feature set produced lower D2 F1-scores than the best MORPH3D and FUSED variants. The best INT-based result was obtained by the feed-forward neural network, with an F1-score for D2 of 0.93. In comparison, the best MORPH3D-based model reached an F1-score of 0.94, whereas the best FUSED-based model reached 0.96. This indicates that grayscale-intensity distribution, intensity variation, and local contrast contain diagnostically useful information, but intensity-based features alone do not fully capture the morphological differences between delamination and crack components. The MORPH3D representation improved the D2 F1-score for most classifier families, confirming that three-dimensional shape features provide strong discriminative information to separate delamination from crack components. The best result within this feature group was obtained using the RBF-SVM classifier. This is consistent with the expected physical difference between the two damage types: delamination components are typically more planar and spatially extended, whereas crack components are usually narrower, more localized, or more elongated.

The highest overall performance was obtained for the FUSED representation, which combined morphological and intensity-based descriptors. This confirms that the two-feature groups provide complementary information. The FUSED-RBF-SVM model achieved the highest overall accuracy and the highest F1-score for class D2. It reached an accuracy of 0.99, a precision for D2 of 0.96, a recall for D2 of 0.96, and an F1-score for D2 of 0.96. The FUSED-Bagged Trees model obtained a slightly lower F1-score for class D2, but it achieved the highest ROC-AUC and PR-AUC values. Therefore, the RBF-SVM model can be considered the best classifier when holdout accuracy and crack-class F1-score are prioritized, whereas the bagged-tree model provides the strongest global ranking performance and class-imbalance-aware evaluation.

The complete numerical results are reported in Table 4. The table includes accuracy, precision, recall, F1-score, ROC-AUC and PR-AUC for all evaluated feature-set and classifier combinations. Therefore, the ranking ability of the models is documented numerically without adding separate ROC or Precision-Recall curve figures to the main text.

**Figure 7:**
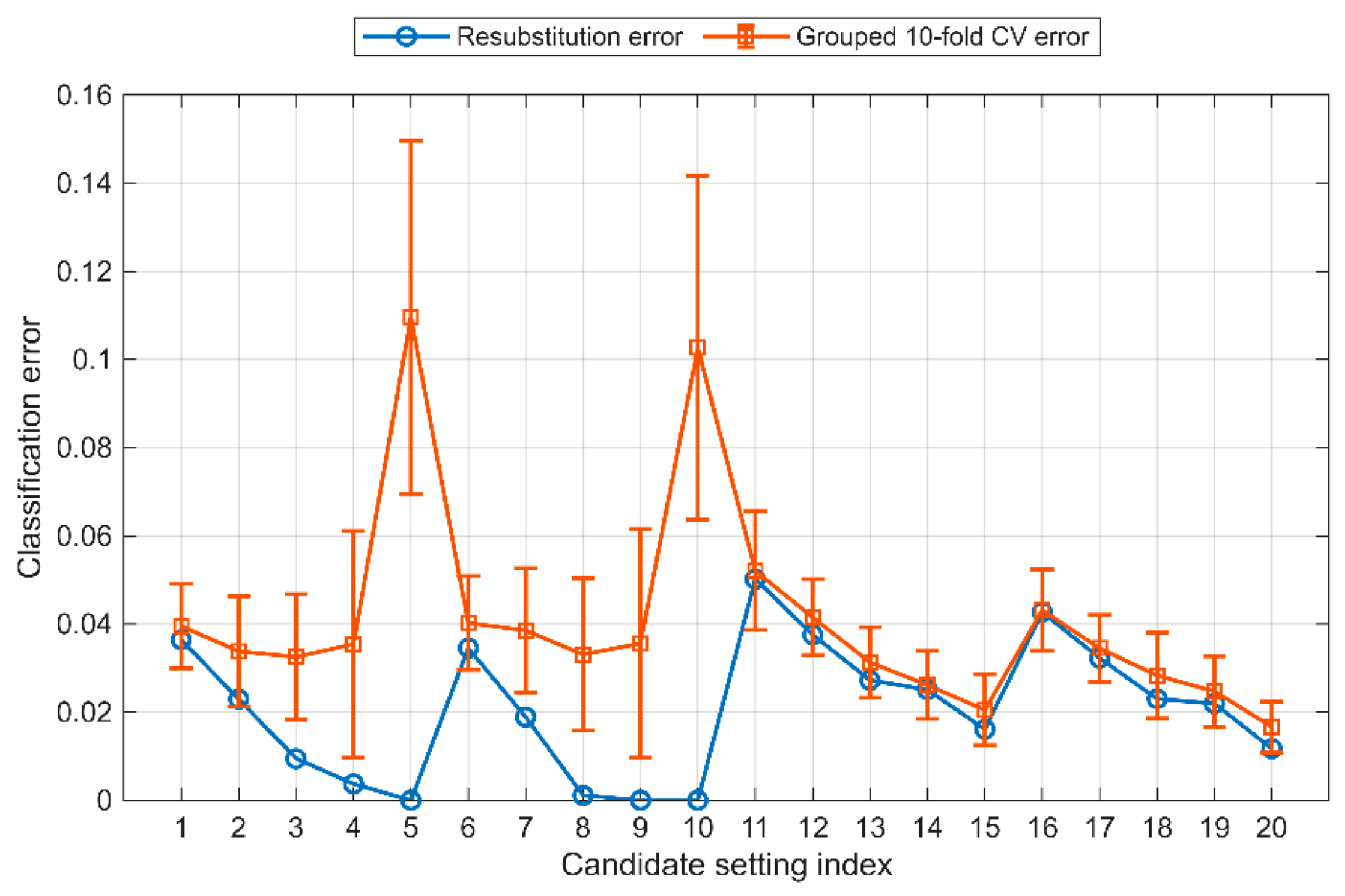
Resubstitution error and mean grouped 10-fold cross-validation error for the evaluated FUSED-RBF-SVM candidate configurations. Error bars denote the standard deviation of validation errors across the 10 grouped folds.

Figure 7 shows the resubstitution and the grouped 10-fold cross-validation errors for the 20 evaluated FUSED-RBF-SVM configurations. For each combination of BoxConstraint ∈ {1, 10} and KernelScale ∈ {1, 10}, models retaining 3, 5, 8, 10 or 24 features were evaluated. The error bars represent the standard deviation of the validation errors across the 10 grouped folds. The lowest mean grouped cross-validation error was obtained for BoxConstraint = 10, KernelScale = 10 and all 24 FUSED features. Therefore, the selected model represents the best-performing candidate within the predefined grid rather than a continuous optimum of the hyperparameters.

The confusion matrix for the best-performing FUSED-RBF-SVM model is presented in Table 5. The model correctly classified 1372 out of 1381 delamination components and 217 out of 227 crack components in the independent holdout subset. Only 9 delamination components were misclassified as cracks, whereas 10 crack components were misclassified as delamination. The row-wise classification accuracy was, therefore, 99.4% for delamination and 95.59% for crack. This confirms that the model maintained high sensitivity to the minority crack class despite the class imbalance.

The remaining FUSED classifiers also produced high-quality classifications, but with slightly different error characteristics. The FUSED-Bagged Trees model produced 12 false crack predictions for delamination components and 9 false delamination predictions for crack components. The FUSED-kNN model produced 14 and 9 such errors, respectively. The shallow rule tree had the highest number of misclassifications among the FUSED models, but it remains important from the interpretability perspective because it provides explicit threshold-based decision rules. Therefore, the rule tree should be treated as a diagnostic model supporting interpretation, whereas the RBF-SVM and bagged-tree models provide the strongest predictive performance.

The feed-forward neural network also achieved high classification accuracy for all three feature representations. Its best result was obtained for the FUSED feature set, with an accuracy of 0.98 and an F1-score for class D2 of 0.94. However, the neural network classifier was less accurate than the FUSED-RBF-SVM and FUSED-Bagged Trees models. Moreover, because the feed-forward neural network is a non-linear model with greater fitting flexibility, its results should be interpreted with caution. In this study, the network was used only as a tabular classifier of previously extracted component features, not as an image segmentation model, and not as a replacement for the 3D U-Net segmentation approach.

In general, the classification results demonstrate that the fused representation of the three-dimensional morphology and the XCT intensity statistics provides the most effective basis for distinguishing the delamination from the crack components. The best-performing models achieved high accuracy, high crack-class F1-score, and high PR-AUC values, confirming that object-level features can support reliable classification of internal fatigue-damage types in XCT data of thermomechanically degraded composite materials.

### 4.4. Expert-Based Labeling for Segmentation

For the segmentation task, an expert-based labeling procedure was introduced to obtain reference annotations independently of the initial image-processing workflow introduced in [28]. Labeling was performed by an expert in XCT inspection and diagnostics of composite materials. A total of 10 expert-labeled two-dimensional XCT slices from five of the six independent holdout cases were used for the expert-based evaluation: CCT-70-1, CCT-80-4, CCT-90-3, CCT-100-2, and CCT-110-1. The slices were purposively selected by an expert with extensive experience in XCT inspection and diagnostics of composite materials to provide diagnostically informative examples across different levels of fatigue degradation and different through-thickness positions. Particular attention was paid to slices containing physically interpretable crack-like regions, because D2 represents the sparsest and most challenging damage class. All 10 selected slices contained expert-identified D2 regions, whereas nine of them also contained D1 regions. The expert annotations were prepared independently of algorithmic reference masks and without access to 3D U-Net predictions. Therefore, the two reference-label sources were used for different purposes. The algorithmic 3D masks provided full-volume supervision and enabled volumetric holdout evaluation, but their results primarily reflect agreement with the deterministic preprocessing workflow from which these masks originated. In contrast, the expert-labeled holdout slices provided an independent local reference for assessing agreement with expert interpretation of physically meaningful D1 and D2 regions.

**Figure 8:**
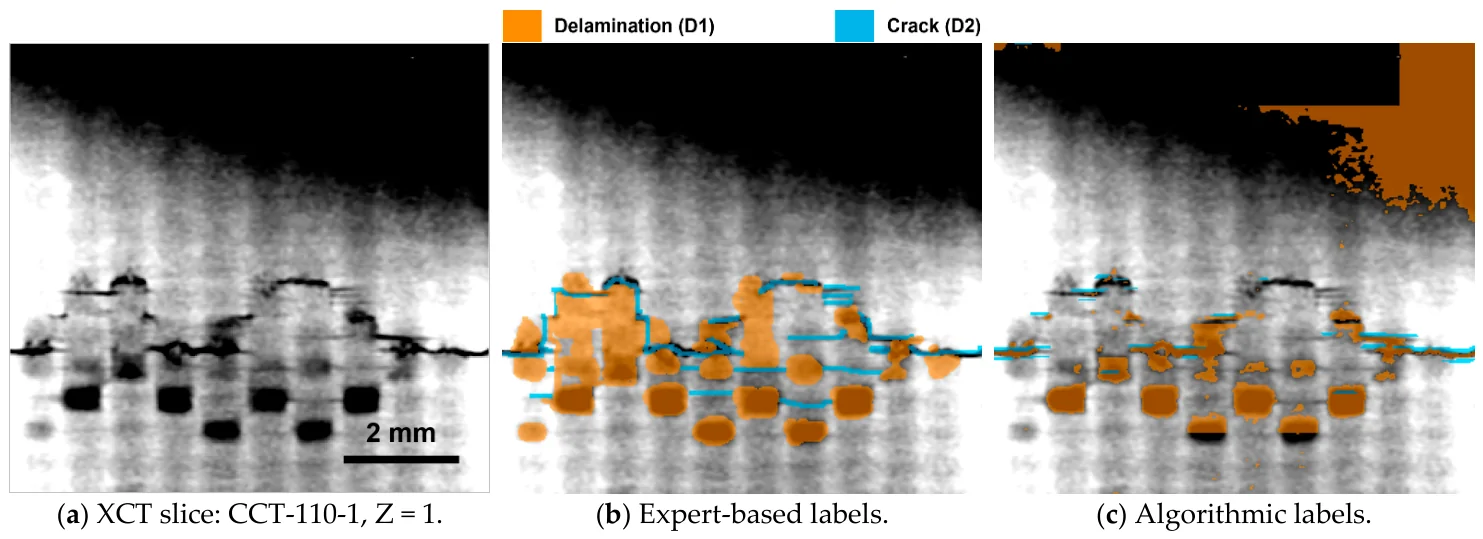

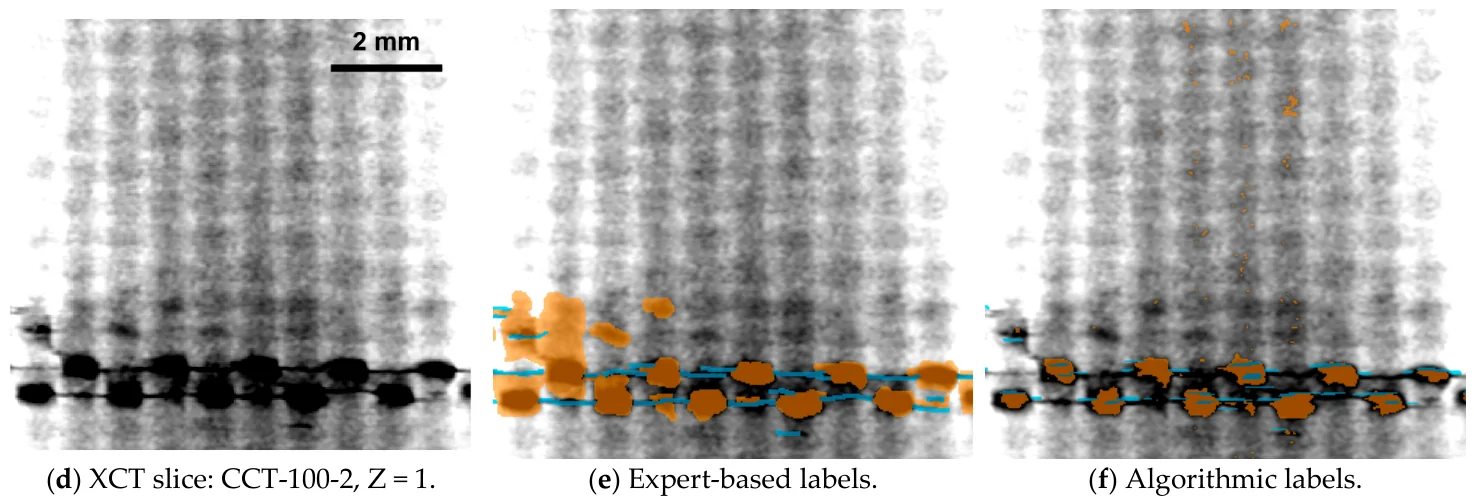
Examples of expert-based ground-truth labeling for selected holdout XCT slices and comparison with labels from the initial image-processing workflow.

Figure 8 presents representative holdout XCT slices with expert-based annotations and the corresponding algorithmic labels. Original XCT slices are shown in Figure 8a,d, the expert-based ground-truth annotations are presented in Figure 8b,e, and the automated masks obtained using the procedure from [28] are shown in Figure 8c,f. The comparison shows that expert-based labels are more selective and better constrained to physically interpretable damage regions.

This difference is particularly important for D1, because these regions may appear in XCT slices as broad, low-contrast areas with boundaries that are not always sharply defined. Therefore, the expert did not assign D1 labels solely on the basis of local grayscale intensity, but also considered the spatial continuity of the damaged region, its relation to the main damage band, and its morphological consistency with delamination-type degradation. For D2, expert annotations were assigned mainly to thin and elongated discontinuities consistent with the visible XCT morphology. Their spatial relation to the D1 regions was also considered, because crack-like structures frequently occur within or near delamination-related damage bands. In contrast, the algorithmic labels may include additional D1 or D2 responses caused by local intensity variations, reinforcement texture, specimen-boundary effects, or XCT artifacts. This is especially visible outside the main damage band, where the deterministic procedure produces isolated or extended false-positive D1 regions and, in some cases, fragmented D2-like responses.

Such algorithmic responses may have grayscale or local geometric characteristics similar to actual damage, but are not necessarily consistent with the expected morphology and location of fatigue-induced D1 and D2 regions.

### 4.5. Segmentation Results

The voxel-wise segmentation results were evaluated according to the multiclass volumetric formulation introduced in Section 2.2 and implemented using the 3D U-Net workflow described in Section 3.3.2. The network was trained to assign each voxel to one of three classes: background (D0), delamination (D1) or crack damage (D2). Since the two damage classes occupied only a small fraction of the XCT volumes, the evaluation was performed separately for each class and was not limited to global accuracy. This was particularly important for the crack-like class D2, which was the rarest class in the analyzed volumes. The training process was monitored using the combined loss function defined in Equation (30), which included weighted cross-entropy and foreground-oriented Tversky loss. The initial training loss was approximately 0.64, whereas the initial validation loss was approximately 0.63. During optimization, both losses decreased substantially and reached final values close to 0.26 after 500 epochs. The lowest validation loss was approximately 0.24. This indicates that the network learned a stable voxel-wise representation of the damage classes. However, the interpretation of the loss values should account for the stochastic nature of patch-based training, the strong class imbalance, and the small number of validation cases.

The class distribution in the training data confirmed the highly imbalanced character of the segmentation task. Background voxels represented 98.29% of the training data, whereas D1 and D2 represented only 1.37% and 0.34%, respectively. To compensate for this imbalance, class weights were introduced in the loss function. The final class weights were 0.0783 for background, 0.6625 for D1 and 2.2592 for D2. Therefore, the contribution of foreground voxels, especially crack-like damage, was increased during optimization. In addition, expert-labeled two-dimensional training slices were incorporated into the training procedure using additional 3D patches passing through manually annotated slices. This resulted in 800 regular training patches and 132 additional expert-guided patches per epoch.

The final evaluation was performed for both raw and post-processed predictions. For each class, TP*_c_*, FP*_c_*, FN*_c_*, and TN*_c_* were computed in a one-vs-rest manner and the *Precision*_c_, *Recall*_c_, F1_c_, *IoU*_c_, and *Dice*_c_ metrics were calculated according to Equations (10)–(12), (37) and (38). In addition to per-volume holdout metrics, global metrics were obtained by summing voxel-level confusion counts over all holdout volumes. Expert-labeled two-dimensional holdout slices were evaluated independently of full-volume algorithmic masks, providing an additional assessment of segmentation quality with respect to manually corrected reference annotations.

Table 6 and Table 7 present the class distributions used in the segmentation evaluation. Table 6 refers to the algorithmic 3D reference labels used for the full-volume holdout evaluation. In this dataset, background voxels constituted 98.39% of all voxels, whereas D1 and D2 constituted only 1.34% and 0.26%, respectively. This confirms that the full 3D evaluation was dominated by the background class and that both damage classes were sparse. Table 7 refers to expert-labeled 2D holdout slices. In this case, the relative amount of damage was higher: D1 represented 3.305% of the pixels, and D2 represented 1.431% of the pixels. This difference results from the fact that the expert evaluation was performed on selected manually annotated slices rather than on complete 3D volumes. Therefore, Table 6 and Table 7 describe complementary reference domains and should not be interpreted as identical test sets.

The quantitative segmentation performance obtained using algorithmic 3D reference labels is reported in Table 8. For the background class, both raw and post-processed predictions achieved very high scores, with F1-score and Dice values close to 0.993. However, this result should be interpreted with caution because the background class strongly dominated the voxel distribution. For D1, the raw prediction achieved Precision = 0.5843, Recall = 0.5058, F1-score = 0.5422, IoU = 0.3719 and Dice = 0.5422. After post-processing, the precision of D1 increased to 0.6104, but the recall decreased to 0.4311, resulting in a lower F1-score of 0.5053. This indicates that post-processing removed part of the false-positive D1 response, but at the cost of removing some true-positive D1 voxels as well.

For D2 evaluated against algorithmic 3D reference labels, the raw prediction was achieved: *Precision* = 0.6239, *Recall* = 0.3783, F1-score = 0.4710, *IoU* = 0.3080 and *Dice* = 0.4710. After post-processing, the precision slightly increased to 0.6360, while the recall decreased to 0.3685. The resulting F1-score and *Dice* were 0.4666. This confirms that the postprocessing stage acted conservatively; it improved the reliability of detected crack-like responses, but did not substantially improve their completeness. The relatively low recall for D2 reflects the difficulty of detecting sparse, thin, and low-contrast crack-like structures in volumetric XCT data.

The results obtained using expert 2D reference labels are summarized in Table 9. Foreground scores were substantially lower than those obtained against the algorithmic 3D references, indicating a reduced agreement when the network predictions were assessed against an independent expert reference. This difference should be interpreted together with the greater selectivity of the expert annotations described in Section 4.4. For D1, the raw prediction achieved *Precision* = 0.3160, *Recall* = 0.4480, F1-score = 0.3706, *IoU* = 0.2275 and *Dice* = 0.3706. After post-processing, the D1 precision increased substantially to 0.4684, while the recall remained nearly unchanged at 0.4465. Consequently, the D1 F1-score increased to 0.4572, and the *IoU* increased to 0.2963. This indicates that the post-processing stage was beneficial when the model output was assessed against manually corrected D1 annotations.

For D2 evaluated against expert 2D reference labels, the raw prediction achieved *Precision* = 0.3393, *Recall* = 0.1587, F1-score = 0.2163, *IoU* = 0.1213 and *Dice* = 0.2163. After post-processing, precision changed only slightly to 0.3461, whereas the recall decreased to 0.1517, resulting in an F1-score = 0.2109 and an *IoU* = 0.1179. These results indicate that crack-like damage remained the most difficult class for the 3D U-Net. The low recall suggests that a considerable part of the expert-labeled D2 pixels was not recovered by the network. This may be attributed to the very small spatial extent of cracks, their weak contrast in individual XCT slices, and their morphological similarity to local texture or acquisition artifacts.

The comparison between Table 8 and Table 9 also shows that the apparent segmentation quality depends strongly on the reference-label source. Algorithmic 3D masks provide complete volumetric supervision and enable full-volume quantitative assessment, but may include systematic responses of the deterministic preprocessing workflow. In contrast, expert labels provide a more selective reference, but only on two-dimensional slices. Therefore, the algorithmic and expert evaluations should be interpreted jointly. The algorithmic evaluation describes how well the 3D U-Net reproduces the full-volume reference masks obtained from the initial image-processing workflow, whereas the expert evaluation indicates how well the same predictions agree with manually corrected damage annotations.

**Figure 9:**
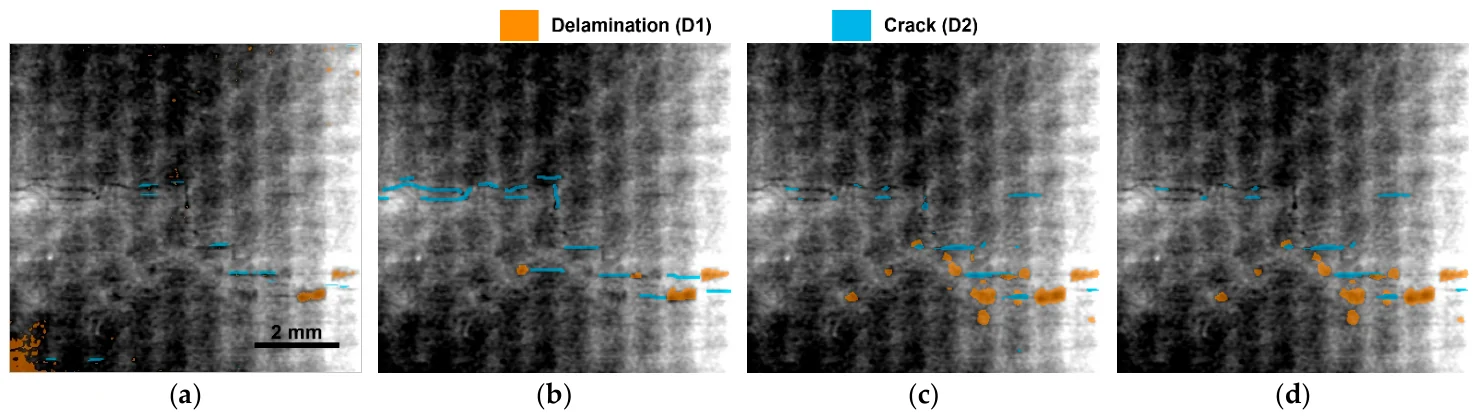
Comparison of algorithmic and expert reference labels with raw and postprocessed 3D U-Net predictions for case CCT-80-4, slice Z = 1. (**a**) Reference labels (algorithm). (**b**) Reference labels (expert). (**c**) Raw 3D U-net. (**d**) Postprocessed 3D U-net.

**Figure 10:**
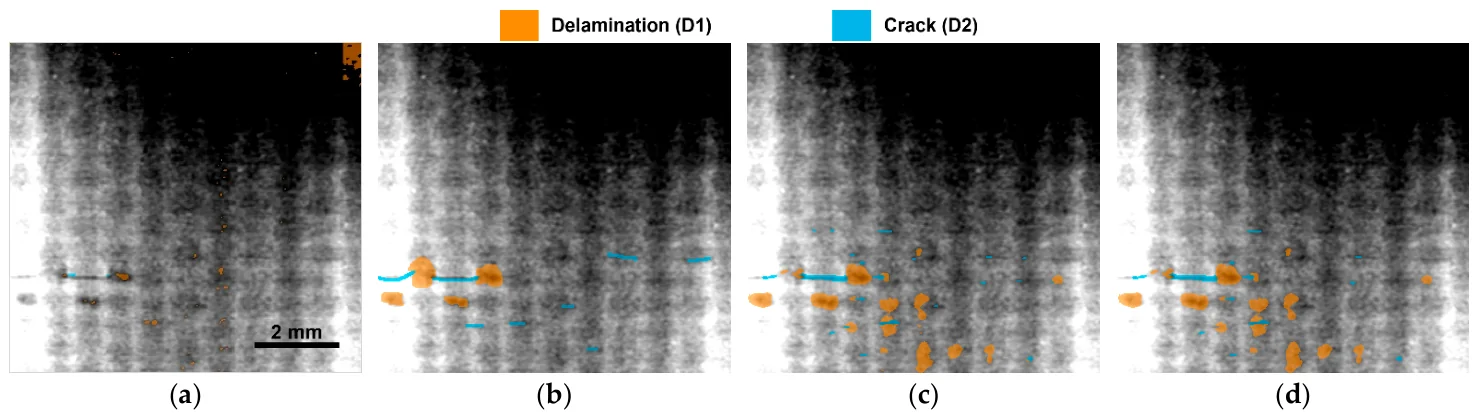
Comparison of algorithmic and expert reference labels with raw and postprocessed 3D U-Net predictions for case CCT-90-3, slice Z = 1. (**a**) Reference labels (algorithm). (**b**) Reference labels (expert). (**c**) Raw 3D U-net. (**d**) Postprocessed 3D U-net.

**Figure 11:**
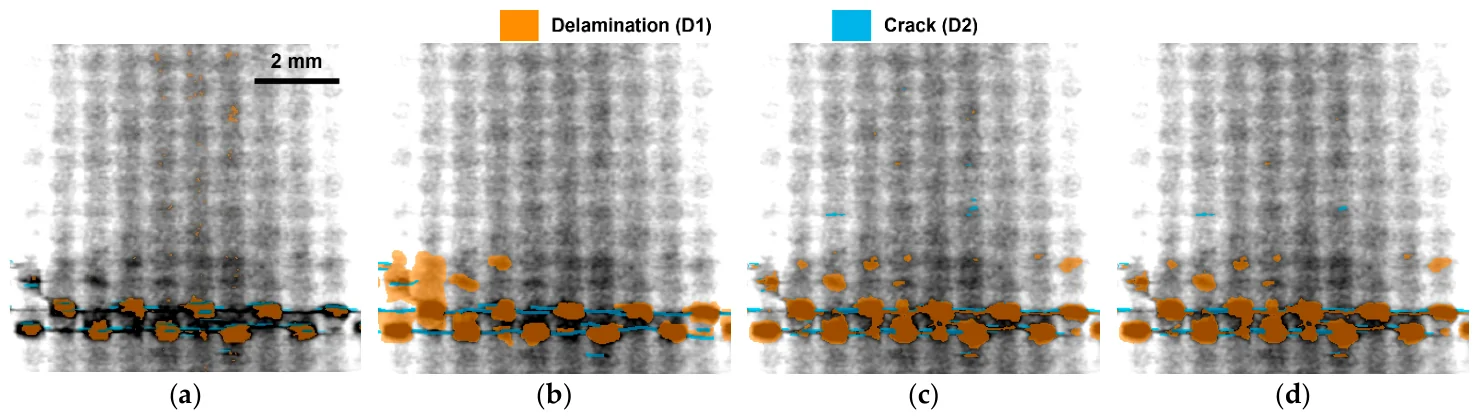
Comparison of algorithmic and expert reference labels with raw and postprocessed 3D U-Net predictions for case CCT-100-2, slice Z = 1. (**a**) Reference labels (algorithm). (**b**) Reference labels (expert). (**c**) Raw 3D U-net. (**d**) Postprocessed 3D U-net.

**Figure 12:**
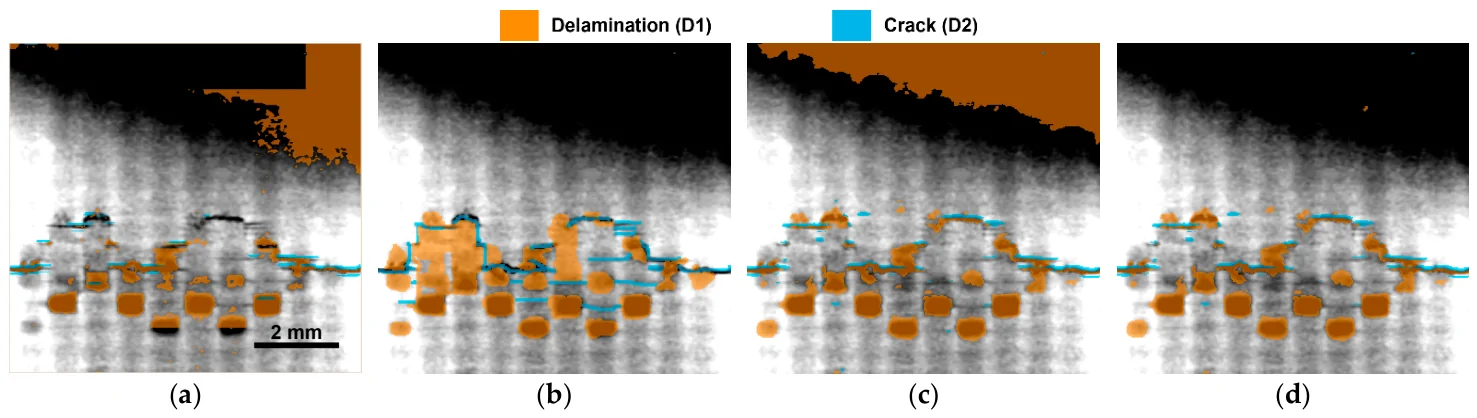
Comparison of algorithmic and expert reference labels with raw and postprocessed 3D U-Net predictions for case CCT-110-1, slice Z = 1. (**a**) Reference labels (algorithm). (**b**) Reference labels (expert). (**c**) Raw 3D U-net. (**d**) Postprocessed 3D U-net.

Representative visual comparisons are presented in Figure 9, Figure 10, Figure 11 and Figure 12 for cases CCT-80-4, CCT-90-3, CCT-100-2, and CCT-110-1, respectively. For visual consistency, the segmentation images in Figure 9, Figure 10, Figure 11 and Figure 12 use the same 2 mm scale bar as in Figure 4 and Figure 8. Each figure compares reference labels obtained from the algorithm, reference labels given by experts, raw 3D U-Net predictions, and postprocessed 3D U-Net predictions. Visual results support the quantitative observations from Table 8 and Table 9. The raw 3D U-Net predictions generally reproduce the main damage regions, but they may also contain additional isolated or spatially extended foreground responses. Post-processed predictions reduce part of these responses and improve the visual compactness of the segmentation, especially for delamination-like regions. At the same time, the visual comparison shows that expert labels are usually more selective than algorithmic labels, which explains why foreground metrics calculated against expert annotations are lower.

The visual observations in Figure 9, Figure 10, Figure 11 and Figure 12 are consistent with the quantitative metrics calculated for the corresponding expert-labeled holdout slices. For CCT-80-4, the post-processed prediction slightly improved the D1 F1-score from 0.4585 to 0.4635, while the D2 F1-score decreased from 0.3374 to 0.3205. For CCT-90-3, the D1 F1-score changed only marginally from 0.4099 to 0.4115 after postprocessing, whereas the D2 F1-score decreased from 0.3121 to 0.3045. For CCT-100-2, the best agreement with expert D1 labels was observed among the presented cases, with the D1 F1-score increasing from 0.6317 to 0.6328 after post-processing. In this case, D2 remained more difficult, with an F1-score close to 0.21. For CCT-110-1, post-processing had the strongest effect on D1, increasing the D1 F1-score from 0.3174 to 0.5578, mainly due to a substantial increase in precision. However, the D2 F1-score remained low and changed only slightly from 0.1978 to 0.1947. These slice-level results confirm that the postprocessing stage mainly improved or stabilized delamination-like damage segmentation, whereas crack-like damage remained the most challenging class. Since these metrics refer to selected two-dimensional expert-labeled slices, they should be interpreted as local quantitative support for the visual comparisons rather than as replacements for the global holdout metrics reported in Table 8 and Table 9.

### 4.6. Discussion of Acquired Results

The feature-based classification results show that object-level features extracted from XCT data can effectively separate delamination and crack components in thermomechanically degraded composite specimens. The comparison between the INT, MORPH3D and FUSED representations indicates that neither intensity-based information nor morphology-based information alone provides the most complete description of the damage components. The best performance was obtained when both feature groups were combined. This confirms that the delamination and crack components differ simultaneously in their three-dimensional geometry and in their local grayscale-intensity characteristics.

The mRMR ranking showed that the most informative descriptors included Wadell sphericity, inscribed-sphere diameter, local contrast, intensity kurtosis, and local contrast-to-noise ratio. These results indicate that the separation between delamination and crack components was not governed only by the size of the components. Instead, classification was influenced by compactness, local thickness, contrast with respect to the surrounding material, and the shape of the intensity distribution. This is consistent with the expected morphology of fatigue-induced damage in polymer-matrix composites. Delamination is usually more planar and laterally extended, whereas cracks may appear as more localized, narrow, or elongated components with different local contrast properties, showing the advantage of the FUSED dataset representation.

The classification results also demonstrate the importance of using metrics appropriate for class-imbalanced data. In the analyzed dataset, crack components represented the minority class. Therefore, the overall accuracy alone would be insufficient to evaluate the practical usefulness of the classifiers. The use of precision, recall and F1-score for class D2, together with PR-AUC, provided a more informative assessment of crack-class recognition. The best model according to holdout accuracy and F1-score for D2 was the FUSED-RBF-SVM classifier, whereas the FUSED-Bagged Trees model achieved the highest ROC-AUC and PR-AUC values. This suggests that the final model choice may depend on the intended diagnostic priority: maximum crack-class F1-score, robust ranking performance, or interpretability.

From an interpretability perspective, the shallow rule tree remains valuable despite its lower predictive performance compared to RBF-SVM and bagged trees. Its explicit threshold-based structure allows the classification decision to be related directly to selected morphological or intensity-based features. Such transparency may be important in non-destructive evaluation, where the diagnostic process should support rather than fully replace expert interpretation. In contrast, RBF-SVM and feed-forward neural networks provide stronger nonlinear classification capability, but their internal decision mechanisms are less transparent. Therefore, a combined use of high-performing nonlinear models and simpler rule-based models may be useful in practical NDE-oriented decision support.

The feed-forward neural network should be interpreted as a classifier of extracted component features, not as a segmentation model. Its high classification accuracy confirms that non-linear relationships between features are present in the feature space. However, its slightly lower performance compared to the best FUSED-RBF-SVM and FUSED-Bagged Trees models, together with its higher fitting flexibility, indicates that neural classifiers should be carefully evaluated under grouped validation and independent holdout testing. In the present workflow, the case-level split, grouped cross-validation, and independent holdout subset were therefore important for obtaining a more reliable performance estimate. Components originating from the same CCT case were kept within the same fold or subset to reduce information leakage, while the training and holdout subsets were selected to preserve a comparable distribution of degradation severity represented by crack and delamination components.

The main limitation of the feature-based classification approach is its dependence on the quality of the previous component extraction stage. Since the classifiers operate on already extracted connected components, segmentation errors, merged components, fragmented objects or reconstruction artifacts may influence the calculated descriptors and, consequently, the final class assignment. This limitation is particularly important in XCT data of polymer-matrix composites, where damage regions may have low contrast and complex three-dimensional morphology. For this reason, feature-based classification should be considered complementary to volumetric segmentation rather than a replacement for it.

The 3D U-Net-based segmentation part of the framework is expected to address a different but related task: voxel-wise identification of damage classes in full XCT volumes. In contrast, the feature-based classifiers discussed here operate at the component level, after the components have been extracted. The final interpretation of the complete workflow should therefore consider both levels of analysis. The segmentation model provides spatially explicit damage maps, whereas feature-based classification provides an interpretable object-level decision stage based on morphological and intensity-based features. Once the segmentation results are fully evaluated, the combined discussion should address whether voxel-wise segmentation and object-level classification produce consistent damage-type interpretation and whether both stages improve the quantitative assessment of thermomechanical fatigue damage.

The obtained segmentation results should be interpreted considering both the quantitative agreement with reference labels and the visual consistency of the predicted damage regions. Voxel-wise metrics provide an objective assessment of segmentation performance, whereas visual comparisons show whether the predicted regions correspond to the spatial morphology of fatigue damage visible in XCT slices. This distinction is important because fatigue-induced damage in polymer-matrix composites is sparse, heterogeneous, and often weakly contrasted. Therefore, a segmentation result may still be diagnostically useful when it correctly localizes the main damaged regions and preserves their morphology, even if the exact voxel overlap with a reference mask is not perfect.

The class distributions confirmed the strongly imbalanced character of the segmentation task. Most voxels represented background or non-damaged material, while delamination-like and crack-like damage occupied only a small fraction of the analyzed volumes. For this reason, high background-related metrics should not be treated as the main indicator of segmentation quality. The practical relevance of the method is better reflected by the class-specific performance for damage classes and by the spatial agreement between reference labels and predictions.

The results obtained for algorithmic three-dimensional reference labels show that the 3D U-Net was able to reproduce the main volumetric damage regions with moderate foreground performance. Raw predictions generally preserved a larger part of the foreground response, whereas post-processing increased the compactness and reliability of the predicted damage regions. This behavior is consistent with the intended role of post-processing, which was designed to remove isolated or artifact-like responses rather than to maximize the total number of detected foreground voxels. Consequently, post-processing should be interpreted as a conservative refinement stage. It improves visual interpretability and reduces spurious detections, but it can also remove weak or small damage fragments. Comparison with two-dimensional expert reference labels provided a stricter assessment of segmentation quality. Expert annotations were more selective than algorithmic masks and limited to regions considered physically interpretable by the annotator. The lower foreground scores obtained against expert annotations reveal a substantial gap between network predictions and independent expert interpretation. This discrepancy was most pronounced for crack-like damage, for which the post-processed expert-based evaluation yielded a recall of 0.1517 and an F1-score of 0.2109. These results demonstrate that reliable segmentation of thin crack-like D2 regions remains a clear limitation of the present model. Differences in the selectivity of the two reference-label sources contribute to the observed performance gap, but do not eliminate the need for further improvement in D2 detection. In this context, the improvement observed for delamination-like regions after post-processing indicates that the conservative refinement stage made the predictions more consistent with the expert interpretation.

Visual analysis supports this interpretation. Raw 3D U-Net predictions generally followed the main damage regions visible in the reference annotations, especially for broader and more continuous delamination-like regions. Post-processed predictions were usually more compact and contained fewer isolated foreground regions, which improved their visual clarity. This is important for practical XCT inspection, where excessive isolated detections may reduce confidence in automated analysis. At the same time, post-processed masks should not be understood as a direct replacement for expert labels. They represent a smooth and conservative volumetric prediction that can support inspection and quantitative analysis. Crack-like damage remained the most difficult class. Crack-like regions are extremely sparse, thin, and often locally ambiguous in XCT data. They may have weak contrast, fragmented geometry and an appearance similar to local texture, reinforcement-related structures, or acquisition artifacts. As a result, the model was less effective in detecting D2 than D1, especially when evaluated against expert annotations. This limitation is expected because detecting thin crack-like structures requires accurate boundary localization and high sensitivity to small foreground regions, while avoiding false positives caused by XCT texture. The obtained results also demonstrate the importance of using both algorithmic and expert reference labels. Algorithmic three-dimensional masks provide full-volume labels and enable volumetric training and evaluation, but they may contain artifacts inherited from deterministic preprocessing. Expert labels provide a more reliable local reference, but they are available only for selected two-dimensional slices. Therefore, neither reference source is sufficient alone. Their combined use gives a more balanced assessment of segmentation quality.

Overall, the proposed 3D U-Net workflow can identify the main volumetric regions of fatigue-induced damage in the XCT data, especially delamination-like damage. The agreement is not perfect at the voxel level, but the predicted regions are generally consistent with the main damage morphology visible in the analyzed data. Therefore, the model should be interpreted as a useful volumetric support tool for damage localization and quantitative analysis, rather than as a fully autonomous replacement for expert inspection. In practical NDE applications, such a tool can reduce manual workload, guide the inspector toward relevant regions, and provide repeatable quantitative descriptors of damage extent. However, the final interpretation should still include expert judgment, particularly for crack-like damage and for regions affected by XCT artifacts or low contrast.

During the development of the segmentation workflow, alternative model configurations, including more complex three-dimensional architectures and different loss formulations, were also explored. These preliminary trials did not indicate a clear benefit of increased architectural complexity and, in several cases, produced lower segmentation performance than the finally adopted 3D U-Net configuration under the investigated development settings. However, these experiments were conducted as part of method development rather than as a controlled comparative benchmark. Therefore, no general conclusion regarding the superiority of the adopted architecture or combined loss formulation can be drawn from these exploratory tests. A systematic comparison of alternative 3D architectures, including Attention 3D U-Net and V-Net, together with a controlled ablation of weighted cross-entropy and Tversky loss components, should be addressed in future work.

## 5. Conclusions

This study presents a comprehensive analysis of the classification of fatigue-induced self-heating damage using the proposed automated approach based on advanced classification and segmentation algorithms, which enables the distinction between delamination and crack damage.

The object-level classification analysis showed that the XCT intensity-based and three-dimensional morphological features provide complementary information to distinguish delamination and crack components. Among the tested INT, MORPH3D, and FUSED representations, the combined FUSED feature set yielded the strongest and most consistent holdout performance, reaching an accuracy of 98.35% for delamination type of damage and 95.59% for cracks, indicating that grayscale-intensity statistics alone do not fully capture the morphology of fatigue-induced damage. The use of CCT-case-level splitting allows obtaining a similar distribution of components with different levels of degradation. These results indicate that interpretable component-level features can complement voxel-wise segmentation by providing quantitative information on damage type, component morphology, and local XCT intensity characteristics.

The 3D U-Net-based segmentation workflow demonstrated that volumetric deep learning can be used to localize the main fatigue-induced damage regions in the XCT data of polymer-matrix composites. The segmentation task was highly class-imbalanced, with many voxels corresponding to background material and only a small fraction representing delamination-like and crack-like damage. Therefore, the obtained results should not only be interpreted through global background-dominated metrics, but mainly through class-specific foreground performance and visual agreement with damage morphology. The model reproduced the main volumetric damage regions with moderate voxel-wise agreement, especially for delamination-like damage with precision 0.61, recall 0.43, F1-score of 0.5, compared to crack damage with the following results: precision 0.63, recall 0.37, F1-score of 0.47. Crack-like damage remained more difficult to segment because of its sparse, thin, and weakly contrasted character. This limitation was particularly evident in the independent expert-based evaluation, where the post-processed D2 predictions achieved a precision of 0.35, recall of 0.15, and F1-score of 0.21. Thus, the reliable detection and delineation of crack-like damage remain the principal limitation of the present segmentation workflow. The comparison between algorithmic 3D reference labels and expert-labeled 2D slices also confirmed that the apparent segmentation performance strongly depends on the adopted reference-label source. Consequently, the proposed 3D U-Net should be regarded as a volumetric decision-support tool for damage localization and quantitative assessment, rather than as a fully autonomous substitute for expert XCT interpretation.

Future work in the segmentation stage should focus on improving the detection of sparse crack-like damage and reducing the dependence of the model on algorithmically generated reference masks. In particular, the preparation of more extensive expert-based three-dimensional annotations, or at least a larger set of expert-labeled slices distributed across different degradation stages, would enable more reliable supervised training and validation. Further improvements may also be achieved by using uncertainty-aware segmentation, attention-based or multi-scale 3D architectures, topology-preserving losses, and semi-supervised or active-learning strategies to exploit unlabeled XCT volumes more effectively. Another important direction is the integration of voxel-wise segmentation with the object-level feature-based classification stage, so that spatially explicit damage maps can be combined with interpretable morphological and intensity-based descriptors. Such a combined workflow could provide more robust quantitative indicators of damage extent, morphology, and evolution, supporting future applications in NDE-oriented diagnostics and residual-life assessment of composite structures. From a practical NDE perspective, the proposed framework is intended as a decision support tool for XCT-based inspection. In particular, such tools can support inspectors in making decisions regarding the presence and type of damage, thereby contributing to improved reliability and greater automation of structural maintenance processes within the NDE 4.0 concept. Once the proposed concept is integrated into an appropriate damage model that links NDT results with an assessment of damage progression, it can also provide information that could be used to estimate the remaining service life of the investigated material or component. The main practical limitation of the proposed approach is its limited scalability to conditions different from those considered in the present study, including the specific material, the loading regime, the damage mechanism, the component thickness, and the XCT acquisition conditions, since these factors can affect damage morphology, the image contrast, the noise, and the artifact levels.

## Figures and Tables

**Table 1:** Distribution of XCT damage components used in the feature-based classification analysis.

Subset	CCT Cases	Components	Delamination, D1	Crack, D2
Training	47	11,149	9014	2135
Holdout	6	1608	1381	227
Total	53	12,757	10,395	2362

**Table 2:** Feature sets used in the classification study.

Feature Set	Number of Features	Feature Type
INT	11	XCT intensity-based and statistical features
MORPH3D	13	Three-dimensional morphological features
FUSED	24	Combined INT and MORPH3D features

**Table 3:** Highest-ranked features for the FUSED representation according to the mRMR ranking.

Rank	Feature	Feature Group	mRMR Score	Interpretation
1	WadellSphericity	Derived morphological ratio	0.294	Differentiates compact components from elongated or planar damage regions
2	InscribedSphereDiameter	Morphological/geometrical	0.264	Describes the local thickness or compact core size of the component
3	LocalContrast	Intensity-based	0.237	Quantifies contrast between the component and the surrounding material
4	IntensityKurtosis	Statistical intensity descriptor	0.171	Describes concentration and extreme values in the intensity distribution
5	LocalCNR	Derived intensity ratio	0.102	Describes component visibility with respect to local intensity variation
6	PrincipalAxisLength2	Morphological/geometrical	0.093	Represents the intermediate spatial dimension of the component
7	IntensityP05	Intensity-based	0.069	Describes the lower tail of the component intensity distribution
8	PrincipalAxisLength1	Morphological/geometrical	0.063	Represents the largest spatial dimension of the component
9	LocalBackgroundStd	Statistical intensity descriptor	0.063	Describes local background heterogeneity around the component
10	IntensitySkewness	Statistical intensity descriptor	0.060	Describes asymmetry of the intensity distribution

**Table 4:** Classification performance on the independent holdout subset. Precision, Recall and F1-score are reported for class D2, corresponding to crack damage.

Feature Set and Model	Accuracy	Precision D2	Recall D2	F1-Score D2	ROC-AUC	PR-AUC
INT—Rule tree	0.96	0.83	0.90	0.86	0.962	0.92
INT—Bagged trees	0.98	0.91	0.93	0.91	0.995	0.97
INT—RBF-SVM	0.97	0.88	0.95	0.91	0.994	0.96
INT—kNN	0.97	0.91	0.89	0.90	0.990	0.96
INT—Feed-forward NN	0.98	0.92	0.93	0.93	0.996	0.97
MORPH3D—Rule tree	0.97	0.90	0.91	0.91	0.978	0.93
MORPH3D—Bagged trees	0.98	0.92	0.92	0.92	0.996	0.98
MORPH3D—RBF-SVM	0.98	0.94	0.94	0.94	0.997	0.98
MORPH3D—kNN	0.98	0.92	0.91	0.92	0.991	0.97
MORPH3D—Feed-forward NN	0.98	0.92	0.96	0.94	0.997	0.99
FUSED—Rule tree	0.97	0.89	0.93	0.91	0.971	0.83
FUSED—Bagged trees	0.99	0.95	0.96	0.95	0.998	0.99
FUSED—RBF-SVM	0.99	0.96	0.96	0.96	0.997	0.99
FUSED—kNN	0.99	0.94	0.96	0.95	0.991	0.98
FUSED—Feed-forward NN	0.98	0.93	0.96	0.94	0.996	0.98

**Table 5:** Confusion matrix for the best-performing FUSED-RBF-SVM model on the independent holdout subset. Percentages are calculated row-wise.

True Class	Predicted D1: Delamination	Predicted D2: Crack
D1: Delamination	1372 (99.35%)	9 (0.65%)
D2: Crack	10 (4.41%)	217 (95.59%)

**Table 6:** Class distribution in the algorithmic 3D reference labels used for the holdout evaluation.

Class	Label ID	Voxel Count	Share (%)
D0	0	54,720,461	98.392
D1	1	746,844	1.343
D2	2	147,315	0.265
Total	-	55,614,620	100.000

**Table 7:** Class distribution in the expert 2D reference labels used for the holdout-slice evaluation.

Class	Label ID	Pixel Count	Share (%)
D0	0	977,499	95.264
D1	1	33,915	3.305
D2	2	14,686	1.431
Total	-	1,026,100	100.000

**Table 8:** Global 3D U-Net segmentation performance on the independent holdout subset using algorithmic 3D reference labels.

Stage	Class	*Precision*	*Recall*	F1-Score	*IoU*	*Dice*
Raw	D0	0.9918	0.9947	0.9932	0.9866	0.9932
	D1	0.5843	0.5058	0.5422	0.3719	0.5422
	D2	0.6239	0.3783	0.4710	0.3080	0.4710
Post	D0	0.9908	0.9959	0.9933	0.9868	0.9933
	D1	0.6104	0.4311	0.5053	0.3381	0.5053
	D2	0.6360	0.3685	0.4666	0.3043	0.4666

**Table 9:** Global 3D U-Net segmentation performance on manually annotated expert holdout slices using expert 2D reference labels.

Stage	Class	*Precision*	*Recall*	F1-Score	*IoU*	*Dice*
Raw	D0	0.9737	0.9674	0.9705	0.9428	0.9705
	D1	0.3160	0.4480	0.3706	0.2275	0.3706
	D2	0.3393	0.1587	0.2163	0.1213	0.2163
Post	D0	0.9739	0.9837	0.9788	0.9585	0.9788
	D1	0.4684	0.4465	0.4572	0.2963	0.4572
	D2	0.3461	0.1517	0.2109	0.1179	0.2109

## Data Availability

The original contributions presented in this study are included in the article. Further inquiries can be directed to the corresponding author.
